# Structural Variant benchmarking frameworks: Parameterization, matching logic, and evaluation assumptions presented through HG002 and NA12878

**DOI:** 10.1371/journal.pcbi.1014824

**Published:** 2026-09-23

**Authors:** Gamze Maden, Mehmet Baysan, Nizamettin Aydın

**Affiliations:** 1 Department of Computer Engineering, Faculty of Computer and Informatics Engineering, Istanbul Technical University, Istanbul, Türkiye; 2 Department of Computer Engineering, Turkish Air Force Academy, National Defence University, Istanbul, Türkiye; University of Helsinki: Helsingin Yliopisto, FINLAND

## Abstract

Structural Variants (SVs) constitute a significant yet complicated class of genomic changes. Due to their imprecise breakpoints, variable size, genomic context and representations across SV callers complicate their accurate detection. Several SV callers have been developed over the years; however, the process of evaluating the outputs of SV callers is inherently more complicated than single nucleotide polymorphisms (SNPs). Unlike SNPs where there is simply a binary representation of whether the nucleotide at a specific position exists, SV analysis must clarify partially overlapping events caused by imprecise breakpoints. This ambiguity has motivated the development of specialized frameworks to support the comparison of SVs. Each of these frameworks implement different assumptions about breakpoint resolution, size concordance or sequence similarity. In this study, we described the underlying strategy of SV comparison modules of three frameworks: Truvari, EvalSVcallers, and SVbenchmark. The primary focus of our study is to reveal how the implemented parameters of each framework influence matching behavior as well as their evaluation outcomes. The conceptual and algorithmic differences of these frameworks are further illustrated through an empirical example based on the query sets of five callers (Manta, Delly, Lumpy, GRIDSS, Wham) for the HG002 and NA12878 reference samples to stress how distinct benchmarking strategies translate into framework-dependent evaluation outcomes. Thus, the reported metrics demonstrate effects of such differences on reproducibility and interpretation rather than prioritizing performance ranking.

## Author summary

Structural Variants are large-scale DNA changes that can affect diversity and diseases; however, evaluating how accurately these variants are detected is not always straightforward. Different benchmarking frameworks use different matching rules when deciding whether a predicted variant matches a truth variant. Therefore, the same variant call set can receive different performance scores depending on the benchmarking framework and parameter settings used. This study compares three commonly used Structural Variant benchmarking frameworks namely, Truvari, EvalSVcallers, and SVbenchmark based on results from five different variant callers and two well-characterized human reference samples, HG002 and NA12878. The analyses show that both framework selection and parameter settings can alter precision, recall, F1-score, and the classification of individual variants. Additional resampling analyses show that most of these differences are not due to random changes in the evaluated genomic regions or variants, but are rather consistently stable. Variant level comparisons also reveal that discrepancies between frameworks are often related to differences in breakpoint location, variant size, or reciprocal interval overlap, but these features do not explain all discrepancies. These findings highlight the importance of transparent reporting of benchmarking methods and interpretation of performance metrics within the context of the framework used.

## 1. Introduction

Structural Variants (SVs) comprise chromosomal alterations including deletions, duplications, insertions, and inversions, typically defined as variants of at least 50 base pairs [1]. Since early whole-genome studies demonstrated their widespread occurrence and diversity in human populations, the systematic identification of SVs has advanced considerably [2]. SVs affect a substantial fraction of the human genome and contribute more altered nucleotides than single-nucleotide polymorphisms, making them an important source of inter-individual genomic diversity [3–6]. Through effects on gene structure, dosage, and regulatory architecture, SVs have been associated with a wide range of inherited and complex diseases, including cancer, autism, Parkinson’s disease, and neurodevelopmental disorders [7–10]. They also contribute to phenotypic diversity, population-specific variation, and evolutionary processes [11–16].

Despite their biological relevance, SVs remain more difficult to detect, represent, and compare than SNPs and small insertions/deletions [17]. SVs may have imprecise breakpoints, heterogeneous representations across callers, complex allelic structures, and variable detectability across repetitive or low-mappability genomic regions [3,4,18–22]. Although advances in long-read and single-molecule sequencing have improved the detection of complex SVs and the construction of benchmark datasets [23–27], factors such as SV size, tandem-repeat context, reference genome choice, truth-set definition, and evaluation strategy continue to influence reported results [28–33]. Consequently, comparisons across SV studies depend not only on the underlying variant calls but also on how those calls are represented and evaluated.

SV callers address these challenges using different sources of sequencing evidence, including discordant read pairs, split-read alignments, read-depth variation, and local assembly [34–47]. Because these strategies differ in their sensitivity to breakpoint uncertainty, SV size, variant type, and genomic context, no single caller consistently performs best across all SV classes and evaluation conditions [48–53]. These caller-specific differences make reliable benchmarking essential, yet benchmarking itself introduces another methodological layer. Previous studies have shown that reported caller performance can vary according to the evaluation methodology, matching criteria, and parameter settings used to compare query and truth sets [54–58].

SV benchmarking frameworks therefore act as more than passive metric calculators. Their assumptions regarding breakpoint proximity, size concordance, reciprocal overlap, sequence similarity, and other equivalence criteria determine which variants are considered matches and consequently affect the assignment of true positives, false positives, and false negatives [59,60]. Different frameworks may therefore assign different classifications and performance metrics to the same query and truth sets. Transparent reporting of comparison logic and parameterization is consequently important for reproducible interpretation and comparison of SV benchmarking results across studies [61,62].

In this context, systematic examination of benchmarking methodology is necessary to distinguish caller-related variation from framework-dependent evaluation effects. Rather than focusing primarily on ranking SV callers, this study investigates three SV benchmarking frameworks—Truvari, EvalSVcallers, and SVbenchmark—with emphasis on their matching logic, parameterization, and underlying evaluation assumptions. Their behavior is first examined at the methodological level and is subsequently evaluated using five SV callers and two reference samples, HG002 and NA12878. The objective is to determine how framework choice and parameterization influence variant classification and reported precision, recall, and F1-score, and to clarify the implications of these differences for reproducible SV benchmarking.

## 2. General principles of SV comparison

SV benchmarking frameworks operate by comparing a *query* set against a *truth* set. A query set can be the variant list generated by any SV caller while a truth set constitutes a collection of validated SVs that serve as ground truth for benchmarking variant callers. The comparison of query and truth set typically involves the following steps:

- *Candidate pairing* based on genomic proximity.
- *Filtering* based on SV type compatibility.
- *Similarity assessment*, which may include size/sequence similarity or reciprocal overlap.
- *Decision assignment*, labeling variants as true positives (TP), false positives (FP), or false negatives (FN) in case of meeting threshold of every matching criterion.

Each of the steps in the comparison process is controlled through user input parameters. Typically, the default parameter values are utilized by SV benchmarking studies to ensure reproducibility and comparability of the results.

The following sections describe the comparison logic, principal parameters, and evaluation assumptions of Truvari, EvalSVcallers, and SVbenchmark. A simplified deletions (DEL) – insertions (INS) example illustrating the sequential matching decisions of the three frameworks is provided in the Supplementary Information as a conceptual demonstration of their differing comparison logic (S1 Appendix).

The methodological comparison was complemented by an empirical evaluation using five SV callers and two reference samples, HG002 and NA12878. The default parameter values of each framework were used as baseline configurations to reflect typical benchmarking practice, and the influence of alternative parameter settings was subsequently examined through systematic sensitivity analyses. This analysis examined how framework-specific matching rules and parameter choices affect variant classifications, precision, recall, and F1-score when the same harmonized caller and truth set inputs are evaluated across the three frameworks.

## 3. Truvari

### 3.1 Overview

Truvari is one of the most widely adopted SV comparison tools and serves as a reference standard in more than two hundred benchmarking studies [63–67]. It is designed to compare two VCF files and determine matching SV pairs based on configurable similarity criteria while preserving allelic diversity [68]. Truvari enables detailed comparison of the SV query set with respect to the truth set, calculating standard performance metrics precision, recall, and F1-score along with variant lists including true positives, false positives, false negatives. As with all comparison tools, Truvari’s performance depends on the quality of input sets and the appropriateness of matching parameters for the specific analysis context.

### 3.2 Matching strategy

Truvari (bench) performs pairwise matching by first identifying SVs that fall within a specified genomic distance and, incorporates haplotype information to refine candidate matches. When multiple candidates exist, the priority is given to the match with the best score. After identifying candidate pairs, multiple similarity constraints are applied, including breakpoint distance tolerance, size similarity, reciprocal overlap, and sequence similarity of alternative alleles.

This behavior is governed by key parameters such as *--refdist (default: 500 bp)*, which defines the maximum allowed distance between SV breakpoints for candidate pairing; *--pctsize (default: 0.7)*, which requires the relative size difference between variants to fall within a specified similarity ratio; *--pctovl (default: 0.0)*, enforcing a minimum reciprocal overlap between SV intervals; and *--pctseq (default: 0.7)*, which specifies the minimum sequence similarity between alternative alleles. An SV pair is considered a valid match only if all the constraints are satisfied. These parameters collectively define the permissiveness of the comparison. Relaxed thresholds increase recall but may inflate false positives, while strict thresholds favor precision at the cost of missed matches.

## 4. EvalSVcallers

### 4.1 Overview

EvalSVcallers is designed to evaluate SV caller performance across standardized datasets [3]. EvalSVcallers enables comparison of SV query and truth sets based on reciprocal overlap, calculates standard performance metrics, including precision and recall, and reports true-positive and false-positive variant lists. A curated NA12878 reference SV dataset (including long-read and assembly-based datasets) is embedded within EvalSVcallers which also supports custom truth sets as input.

### 4.2 Matching strategy

Evaluation of SV callers in EvalSVcallers involves two phases. First, the output for each caller is converted to a standard form, then filtered by SV type and size restrictions. The determination of a match for the non-insertion SVs is based on the reciprocal overlap between called and reference variants, while insertions are evaluated strictly based upon their positions. The comparison is performed using a predefined truth set and evaluation metrics are computed for each SV type and size class.

This behavior is governed by key parameters such as *--min_ovl (default: 0.5)*, which specifies the minimum required reciprocal overlap between called and reference non-INS SVs for a valid match; *--sv_type (default: ALL)*, restricting the comparison to selected SV classes; *--len* and -*-xlen (default: 50 bp and 2000000 bp)*, defining the minimum and maximum sizes of called SVs; and *--ref_len* and -*-ref_xlen (default: 50 bp and 2000000 bp)*, applying equivalent size constraints to reference SVs. An SV is considered a match if it satisfies the overlap requirement (where applicable) and falls within the defined size and type filters, resulting in a binary matched–unmatched decision without additional breakpoint or sequence-level similarity constraints.

## 5. SVbenchmark

### 5.1 Overview

SVbenchmark is part of the SVanalyzer toolkit and provides an alternative strategy for SV comparison based on haplotype sequence comparison [69]. SVbenchmark enables comparison of the SV query set with respect to the truth set, calculates standard performance metrics, including precision, recall, and F1-score, and reports distance computations as well as false-positive and false-negative variant lists.

### 5.2 Matching strategy

SVbenchmark evaluates SV concordance by reconstructing and comparing alternate haplotype sequences implied by the query and truth sets. Candidate variant pairs are first screened based on maximum distance threshold, after which similarity is evaluated using normalized measures that account for positional shifts, size and sequence differences between alternate alleles.

This behavior is governed by key parameters such as *--maxdist* (default: 100000 bp), which restricts matches to variants whose genomic positions lie within a defined distance; *--normshift* (default: 1.0), which limits the allowable normalized alignment shift between alternate alleles; *--normsizediff* (default: 1.0), which constrains the normalized size difference of the reconstructed alleles; *--normdist* (default: 1.0), which sets the maximum permitted normalized edit distance between alternate haplotype sequences; and *--minsize* (default: 0 bp), which filters variants below a minimum length. An SV is considered a match if the reconstructed alternate haplotypes satisfy all normalized similarity constraints, yielding a binary matched–unmatched outcome based on sequence-level equivalence.

## 6. Comparative perspective

Although the three comparative tools have the same general objective to evaluate SV call sets, they are very different in terms of defining and enforcing what constitutes similarity among calls. As a result, the various evaluation metrics produced by these tools are not compatible and these differences make results difficult to compare, complicating performance assessment and obscuring which results best represent the callers’ true ability to detect SVs accurately. Below we demonstrate the differences based on a single SV that was independently assessed by each comparison metric. Table A and B in S1 Appendix provide the query and truth call set variants as a simple example including their genomic locations, variant type, genotype and haplotype (S1 Appendix). This example illustrates how each benchmarking framework processes the same set of variants and highlights the sequential decision steps leading to their final classifications.

## 7. Materials and methods

The HG002 and NA12878 samples are a well-established reference standard for SV benchmarking due to the availability of high-confidence truth sets derived from several different sequencing technologies and validation strategies. HG002 truth set is part of the Genome in a Bottle (GIAB) consortium and has been broadly characterized by long-read sequencing, assembly-based methods and manual curation, therefore providing a robust and reproducible foundation for evaluating SV benchmarking frameworks. The NA12878 truth set is part of Human Genome Structural Variation Consortium (HGSVC) Freeze 3 release and this high-confidence SV truth set undergoes a rigorous multi-method validation process, including k-mer analysis, sub-sequence alignment, and orthogonal callers, ensuring high-quality variant calls across a diverse genomic landscape (the embedded NA12878 truth set provided with EvalSVcallers was not used in these experiments because its framework-specific structure could not be directly used as a common truth set input across the other benchmarking frameworks). HG002 and NA12878 serve as suitable samples for illustrating framework-specific differences in SV comparison behavior owing to frequent adoption in previous benchmarking studies and comprehensive validation [70–78].

### 7.1 Samples and sequencing data

In this study, for HG002, the approximately 50 × pre-aligned BAM file was obtained directly from the NIST Genome in a Bottle Ashkenazim Trio repository. The reads were aligned to the Human UCSC hg19 reference genome using BWA v0.6.1-r104-tpx-isis within the BaseSpace BWA Whole Genome Sequencing workflow v1.0.0.1. PCR-duplicate flagging was enabled which also incorporated SAMtools v0.1.18 and GATK v1.6-22-g3ec78bd. For NA12878, paired-end whole-genome sequencing reads from SRA accession SRR17658585 were aligned to GRCh38 reference genome using BWA-MEM2 v2.2.1. The resulting alignments were converted to BAM format and coordinate-sorted using SAMtools v1.13. The final BAM contained no reads flagged as duplicate.

### 7.2 SV calling

Structural Variant call sets were generated independently for HG002 and NA12878 using the same caller versions and sample specific reference assemblies. Manta v1.6.0 was configured in diploid mode using the sample specific BAM and corresponding reference FASTA. Delly v1.2.6 was executed in call mode with the corresponding reference genome supplied through the -g option. Lumpy v0.2.13 was run through the lumpyexpress workflow; discordant paired-end and split-read alignments were extracted separately and supplied together with the full BAM, and the resulting calls were genotyped using SVTyper v0.7.1. GRIDSS v2.13.2 was executed using the gridss.CallVariants module with the sample specific BAM and reference genome. Wham v1.7.0-311-g4e8c-dirty was run with the sample specific BAM and reference genome supplied through the -f and -a options, respectively. The resulting caller VCFs were subsequently subjected to the common preprocessing and representation standardization workflow described below.

### 7.3 Data preparation and genomic context standardization

All five caller VCFs (Manta, Delly, Lumpy, GRIDSS, and Wham) were first passed through the OctopuSV v0.3.1 [79] representation-standardization workflow, including the conversion of BND records into standardized SV representations where applicable. Following caller-output standardization, common filtering criteria were applied to both caller and truth set VCFs. The standardized caller outputs and truth sets were subsequently restricted to autosomal chromosomes (chr1–chr22), PASS records, DEL and INS variants, and SVs with an absolute length ≥50 bp. This design ensures that all observed differences in the outputs of the comparisons are a result of the internal comparison logic of each framework rather than from variations in input data. Additionally, variants in HG002 were restricted to the high-confidence benchmark regions defined by the corresponding truth set BED file, thereby limiting the evaluation to the validated benchmark domain. No equivalent high-confidence BED file is currently available for NA12878. Because the genomic evaluation domains differed between the two samples, cross-sample differences are presented only descriptively and should not be interpreted as performance comparisons. Caller-specific record counts before and after OctopuSV standardization and subsequent preprocessing steps are provided in Supplementary Table D in S1 Appendix. The resulting standardized and filtered caller and truth set VCFs were then used as common inputs to the three benchmarking frameworks within each sample.

### 7.4 SV benchmarking

Benchmarking was performed independently for HG002 (GRCh37) and NA12878 (GRCh38), both using approximately 50 × whole-genome sequencing data. For each sample, the caller specific query sets described above were evaluated against the same corresponding truth set using Truvari v3.0.0, EvalSVcallers, and SVbenchmark through SVanalyzer v0.36.

### 7.5 Genomic block resampling

Genomic block resampling analysis was performed to assess how stable the default benchmarking results were across genomic regions. Autosomal chromosomes were divided into separate, non-overlapping 5 Mb genomic blocks for each reference genome/sample combination. Block files were created separately for each sample because NA12878 and HG002 were evaluated using different reference assemblies and chromosome lengths. 200 resampling iterations were generated for each sample. In each iteration, 80% of the autosomal genomic blocks were randomly selected without replacement. Sampling without replacement was used since the classic bootstrap approach could lead to duplicate VCF entries and affect the variant matching process. Therefore, this approach should be interpreted as a bootstrap-inspired genomic block resampling strategy instead of the classic bootstrap. In each resampling iteration, the same selected genomic blocks were applied to both the caller VCF file and the corresponding truth set. Thus, in each iteration, the truth and caller variants were evaluated on the same genomic regions. Each subset was then re-benchmarked using the default configurations of Truvari, SVbenchmark, and EvalSVcallers. This approach aimed to capture the sensitivity of benchmarking metrics to regional genomic composition and framework specific matching behaviors, as it re-runs the variant matching process within each genomic subset. For each sample, the mean, standard deviation, median, and empirical 2.5% to 97.5% percentiles of precision, recall, and F1-score values for the caller and benchmarking framework combination were calculated over 200 resampling iterations.

### 7.6 Baseline TP, FP, and FN characterization

Baseline benchmarking outcomes were additionally characterized at the variant level according to SV type and size. Variants were stratified as DEL or INS and into five size intervals: 50–99 bp, 100–499 bp, 500–999 bp, 1–9.9 kb, and ≥10 kb. FP and FN rates were summarized separately within each sample–caller–framework–SVTYPE–SVLEN stratum.

### 7.7 Parameter sensitivity analysis

Parameter sensitivity was performed using a systematic one-factor-at-a-time (OFAT) design in which one evaluation parameter was varied while all remaining parameters were held at the study baseline. Truvari and SVbenchmark were each evaluated using the baseline configuration and 16 alternative configurations, whereas EvalSVcallers was evaluated using the baseline and 8 alternative configurations. Across two samples and five SV callers, this resulted in a total of 430 sample-caller-framework-configuration evaluations. For Truvari, the baseline configuration was *refdist* = 500, *pctsize* = 0.7, *pctseq* = 0.7, and *pctovl* = 0.0. The OFAT analysis evaluated *refdist* values of 100, 250, 750, and 1000 bp; *pctsize* values of 0.5, 0.6, 0.8, and 0.9; *pctseq* values of 0.5, 0.6, 0.8, and 0.9; and *pctovl* values of 0.2, 0.4, 0.6, and 0.8. For SVbenchmark, the baseline was *maxdist* = 100000, *normshift* = 1.0, *normsizediff* = 1.0, and *normdist* = 1.0, with *minsize* = 0 held fixed. The alternative configurations evaluated *maxdist* values of 1000, 10000, 50000, and 200000 bp and independently varied each normalized criterion across 0.1, 0.3, 0.5, and 0.7. For EvalSVcallers, the baseline matching parameters were *min_ovl* = 0.5 and *min_ins* = 200. The *xlen* and *ref_xlen* parameters were held fixed at 2000000 bp because they define caller-side and truth-side SV-size limits for the evaluated domain rather than the pairwise variant-matching criterion. The OFAT analysis therefore focused on *min_ovl* and *min_ins*, evaluating *min_ovl* values of 0.3, 0.4, 0.6, and 0.7 and *min_ins* values of 50, 100, 300, and 500 bp. Sensitivity was quantified primarily using absolute changes in F1-score relative to the baseline (ΔF1) and parameter-specific F1-score ranges. This approach avoids disproportionately large percentage differences when the baseline F1-score is close to zero.

### 7.8 Variant level bootstrap analysis

Statistical assessment of parameter-associated F1-score changes was performed using paired variant level bootstrap resampling. For each sample-caller-framework combination, caller-side and truth-side variants were resampled independently with replacement in 5000 bootstrap iterations (using the variant-level TP/FP and TP/FN classifications obtained from the original benchmarking results). Caller-side TP/FP outcomes were used to recalculate precision, while truth-side TP/FN outcomes were used to recalculate recall. Within each bootstrap iteration, the same resampled caller indices and the same resampled truth indices were applied to both the baseline and the corresponding alternative configuration. This preserved a paired comparison between parameter settings while allowing the composition of the evaluated variants to vary across bootstrap iterations. A new F1-score was calculated for the baseline and alternative configuration in each iteration, and the difference was defined as ΔF1 = F1_alternative_ − F1_baseline_. The 2.5th and 97.5th percentiles of the 5000 ΔF1 values were used as the empirical 95% bootstrap interval. A parameter-related change was considered bootstrap-supported when this interval did not include zero, indicating that the direction of the F1-score change remained consistent across the resampled variant sets.

### 7.9 Cross-framework variant level characterization

To enable variant level comparison across benchmarking frameworks, a unified query-variant universe was constructed for each sample-caller combination from the union of query-side variant records returned by the three frameworks. Framework-specific TP and FP outcomes were retained for each query variant, whereas variants for which a framework returned no outcome were assigned the label NONE. NONE was used only as a cross-framework bookkeeping category indicating the absence of a reported outcome and does not represent a classification generated by the benchmarking framework. To further characterize the variant properties associated with cross-framework agreement and disagreement, query variants in each unified variant universe were stratified according to the number of benchmarking frameworks that classified the same variant as a TP. Variants were categorized as TP in all three frameworks, TP in exactly two frameworks, or TP in only one framework. For each query variant, the nearest truth-set variant of the same SV type on the same chromosome was identified using absolute start-position distance. Breakpoint displacement was calculated as the absolute difference between query and truth start positions. Size agreement was summarized using the ratio of the smaller to the larger absolute SV length, with values approaching 1 indicating greater size concordance. For DEL variants, reciprocal overlap was additionally calculated as the smaller of the fractions of the query and truth intervals covered by their intersection. INS variants were characterized using breakpoint displacement, absolute size difference, and size ratio. Sequence similarity was not included as a common cross-framework characterization metric because an equivalent framework-selected sequence-similarity measure was not available consistently from all three benchmarking outputs. Using a sequence-similarity value derived from only one framework would make the common characterization dependent on that framework’s internal matching procedure. Similarly, one-to-one, clustered, and potentially one-to-many representation effects were not quantified as a common metric because these behaviors are handled differently internally by the three frameworks and cannot be reconstructed uniformly from their final outputs. Cross-framework TP agreement groups were additionally summarized across the same five query-variant size intervals used for the baseline FP/FN characterization (50–99 bp, 100–499 bp, 500–999 bp, 1–9.9 kb, and ≥10 kb). Categories containing fewer than 10 variants were retained for completeness but were not used for distribution-level interpretation since summary statistics from these small groups may be disproportionately influenced by individual variants. Representative loci were selected from predefined cross-framework agreement patterns to illustrate contrasting forms of concordance and discordance. Within each targeted pattern, the variant closest to the group median characteristics was selected using breakpoint displacement, size agreement, and reciprocal overlap where applicable, with feature distances scaled by their interquartile ranges to prevent differences in numerical scale from dominating the selection.

### 7.10 Reproducibility

All preprocessing, benchmarking, sensitivity, resampling, and post-processing steps were documented at command level to support reproducibility. The repository includes centralized configuration files, truth set and caller preprocessing workflows, framework specific baseline evaluations, systematic parameter sensitivity analysis, genomic block resampling, paired variant level bootstrap analysis, baseline TP/FP/FN characterization, cross-framework variant level characterization, figure and supplementary table generation scripts, environment definitions, and repository level validation utilities. The principal repository components and their roles are summarized in TableC in S1 Appendix (S1 Appendix).

## 8. Results

HG002 and NA12878 query sets from five callers (Manta, Delly, Lumpy, GRIDSS, and Wham) were evaluated against the corresponding truth sets using Truvari, EvalSVcallers, and SVbenchmark. Because each framework applies its own matching strategy and definition of variant equivalence, the resulting performance metrics represent framework-specific interpretations of the same underlying caller and truth set inputs (as shown in S1 Appendix: S1 Appendix. Algorithmic Logic of SV Benchmarking Frameworks). For cross-framework variant level analyses, the unified variant universes defined in the empirical evaluation workflow were used to compare TP, FP, and NONE outcomes assigned to the same query variants across the three frameworks.

### 8.1 Characteristics of the preprocessed benchmarking datasets

Following preprocessing, the HG002 truth set contained a total of 28023 variants (10662 DEL, 17361 INS), whereas the truth set of NA12878 consists of a total of 15296 variants (5698 DEL, 9598 INS). Figs 1 and 2 illustrate distributions of SV types in the query sets compared to HG002 and NA12878 truth sets respectively. Figs 3 and 4 depicts distribution of SV lengths of query sets compared to HG002 and NA12878 truth sets respectively. The box plot summarizes the distribution of absolute SV length (SVLEN) values for both caller-derived query callsets and high-confidence truth sets on a logarithmic scale, showing the median, interquartile range, and outliers. The SVLEN distributions indicate that, for deletions, most callers were located within a broadly similar range to the truth set (log10 ~ 2.0–2.5). Manta showed the closest median to the truth set in both samples, whereas Lumpy, GRIDSS, and Wham showed somewhat higher median deletion lengths. For insertions, the differences were more pronounced: Delly and Lumpy reported substantially larger insertion lengths, with median values extending above the range observed in the corresponding truth sets.

**Fig 1:**
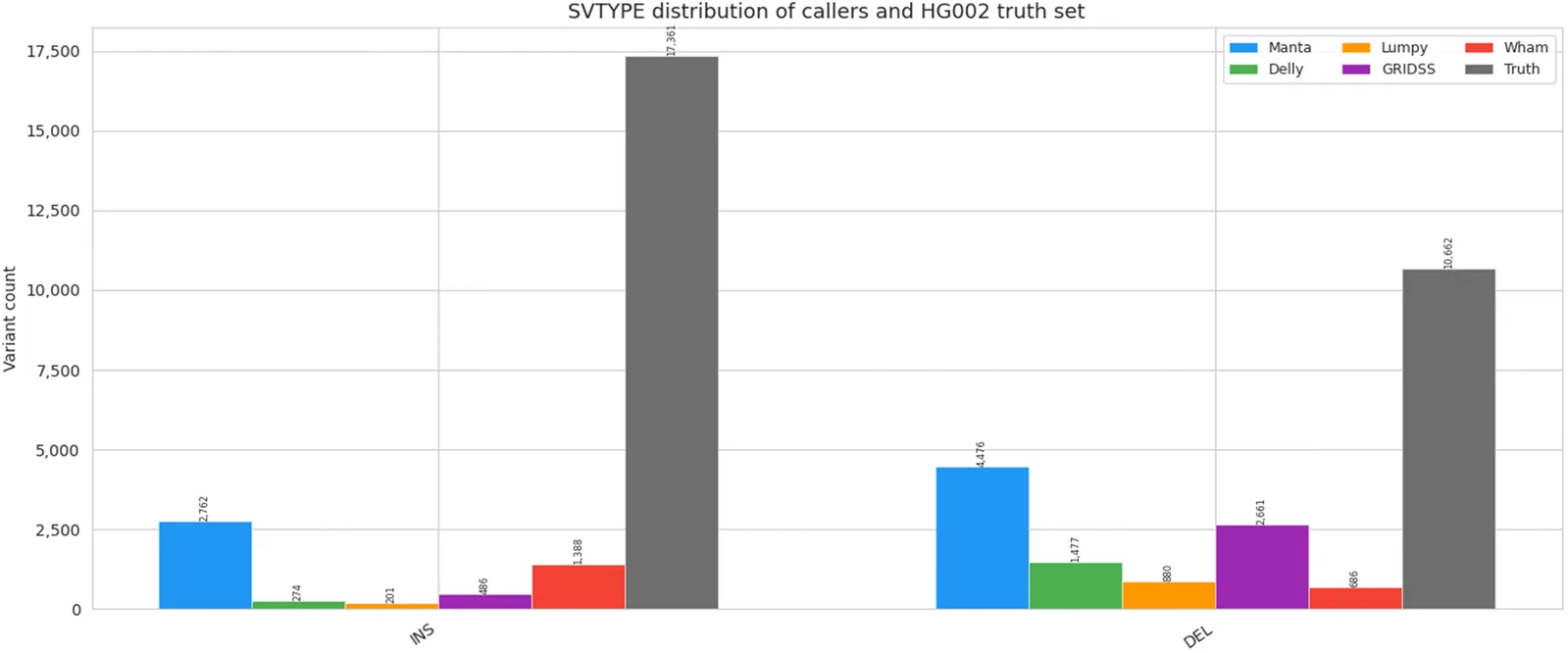
Distribution of SV types (SVTYPE) for HG002.

**Fig 2:**
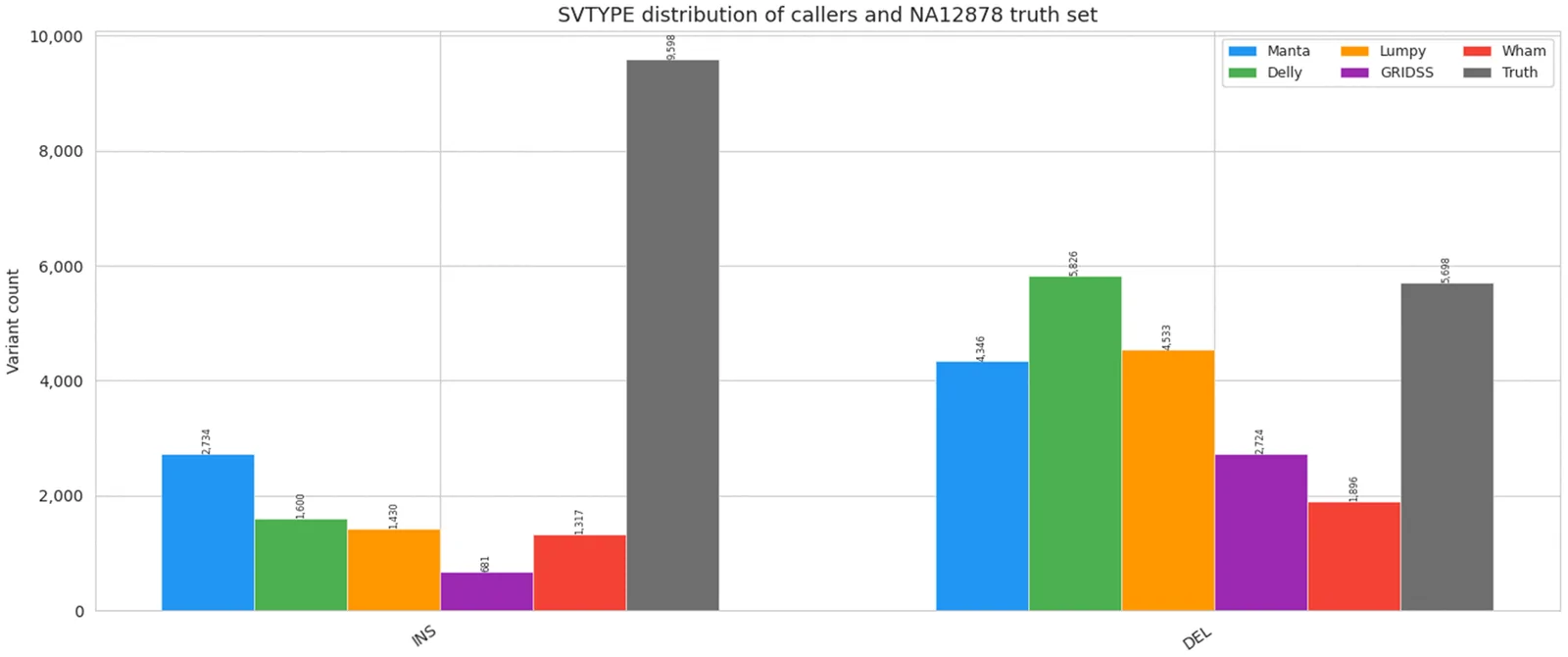
Distribution of SV types (SVTYPE) for NA12878.

**Fig 3:**
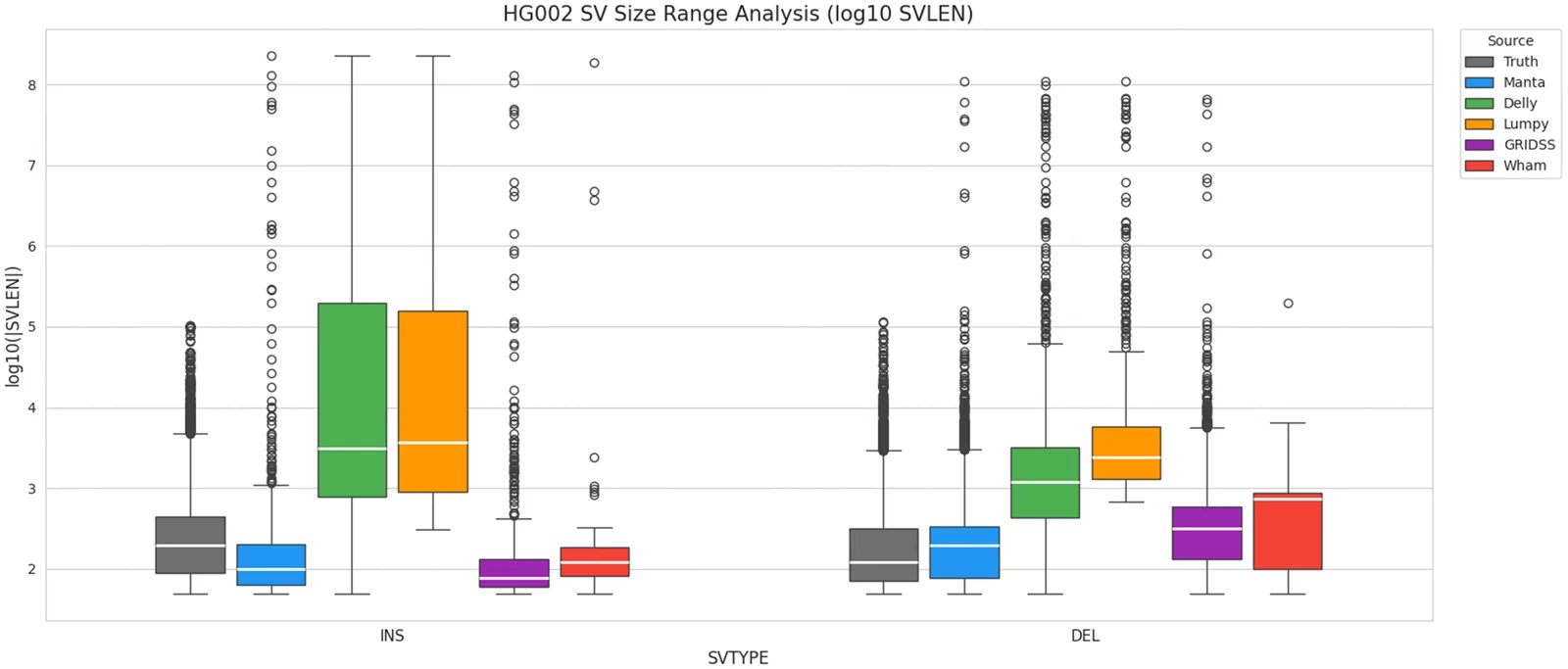
Distribution of SV lengths (SVLEN) for HG002.

**Fig 4:**
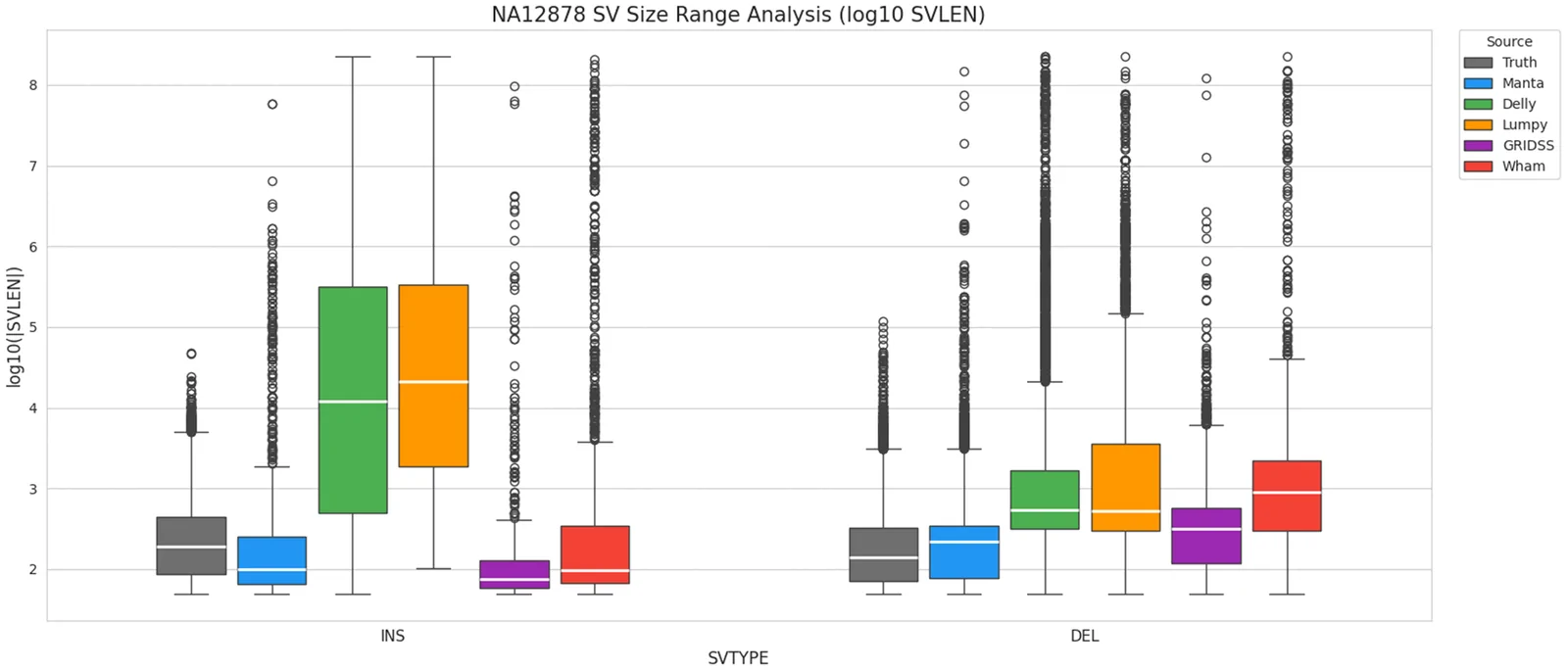
Distribution of SV lengths (SVLEN) for NA12878.

### 8.2 Baseline benchmarking performance

SV calls from Manta, Delly, Lumpy, GRIDSS, and Wham on the HG002 and NA12878 samples were evaluated using the Truvari, EvalSVcallers and SVbenchmark. Fig 5 presents the aggregate bar charts of precision, recall, and F1-score values for each sample-caller-framework combination under default framework configurations. The figure reveals both common performance trends and framework-dependent differences in the absolute metric values assigned to the same caller outputs. In the HG002, precision values generally remained higher than recall values in all frameworks. Specifically, for Manta, precision values were obtained as 0.9015 in Truvari, 0.9315 in EvalSVcallers and 0.9315 in SVbenchmark while recall values remained at 0.2319, 0.0907 and 0.2743 respectively. Similarly, for the other callers, precision was found to be relatively high, while recall was lower leading to medium or low level F1-scores. For example, the F1-score for Manta was calculated as 0.3689 in Truvari, 0.1653 in EvalSVcallers and 0.4238 in SVbenchmark. The same general trend was observed for Delly, Lumpy, GRIDSS, and Wham, but the absolute values of the metrics varied depending on the caller and framework. The same framework-dependent variation in precision, recall, and F1-score was also observed in NA12878. For Manta, precision values were calculated as 0.4344 in Truvari, 0.4718 in EvalSVcallers and 0.4595 in SVbenchmark while recall values were obtained as 0.1976, 0.0890 and 0.2135 respectively. In contrast, F1-scores remained at 0.2717, 0.1498 and 0.2915. Delly, Lumpy, GRIDSS, and Wham results similarly showed limited performance in terms of both precision and recall. In particular, recall values were quite low for Delly and Wham, which significantly reduced F1-scores. Overall, the three frameworks showed broadly similar precision–recall trends across callers, while the absolute precision, recall, and F1-score values assigned to individual caller outputs varied across frameworks.

**Fig 5:**
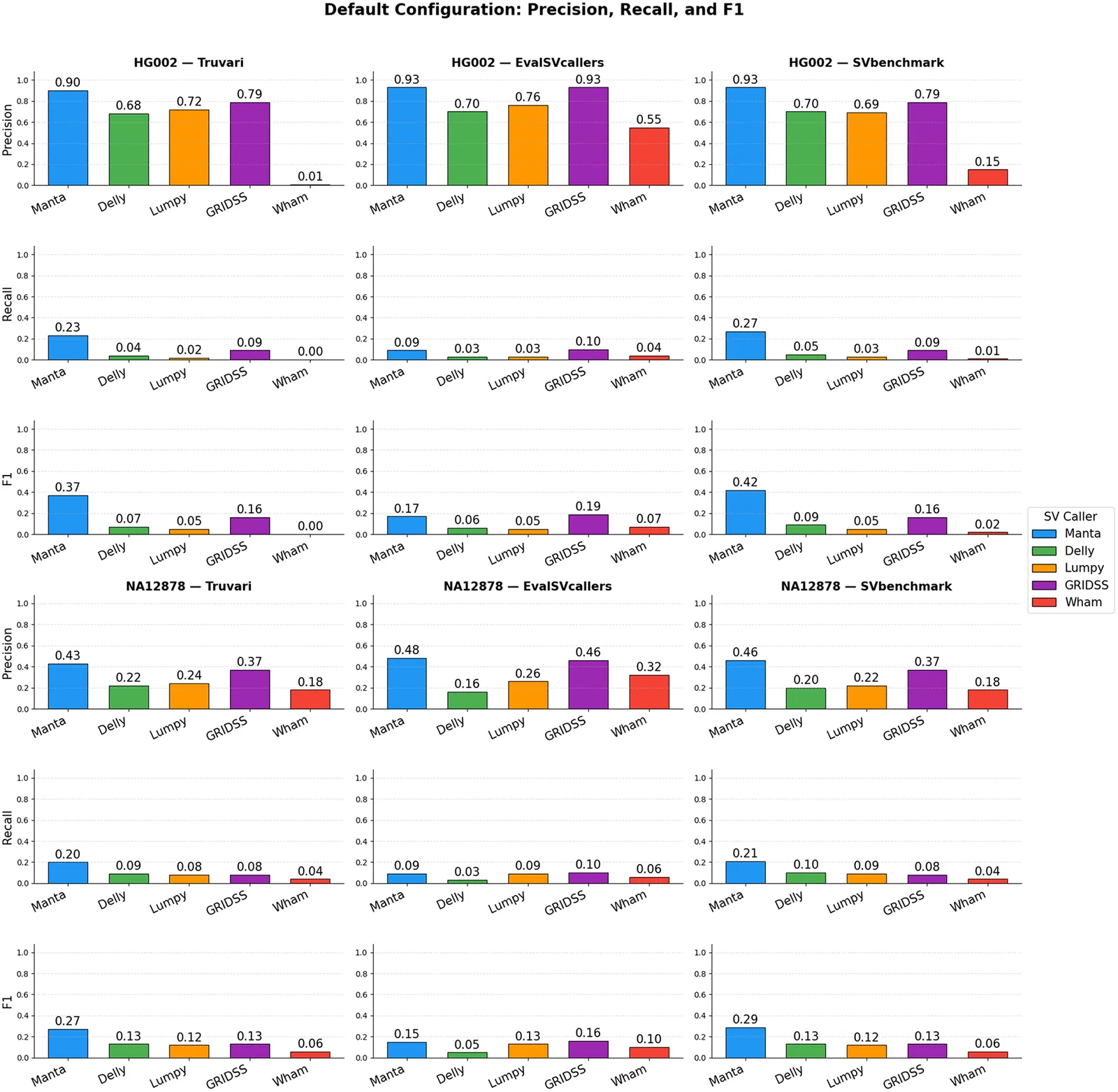
Performance metrics under default configurations for HG002 and NA12878.

### 8.3 SV type specific macro-F1 estimation

Fig 6 provides a type-specific evaluation of the baseline results. In this analysis, DEL and INS F1-scores were calculated separately for each caller and benchmarking framework. Macro-F1 was then calculated as the mean of DEL-F1 and INS-F1. DEL-F1 was generally higher than INS-F1 across most callers and benchmarking frameworks. Under Truvari and Svbenchmark, Manta showed the highest Macro-F1 values in both samples, whereas Delly, Lumpy, GRIDSS, and Wham generally showed lower Macro-F1 values, largely due to limited INS performance. Under EvalSVcallers, however, the highest Macro-F1 was observed for Delly in HG002 and GRIDSS in NA12878. The heatmap also shows that benchmarking framework choice affected the absolute scores assigned to the same caller, although the overall caller ranking was broadly consistent across frameworks. These findings support the interpretation that SV benchmarking results are also influenced by SVTYPE-specific detection behavior.

**Fig 6:**
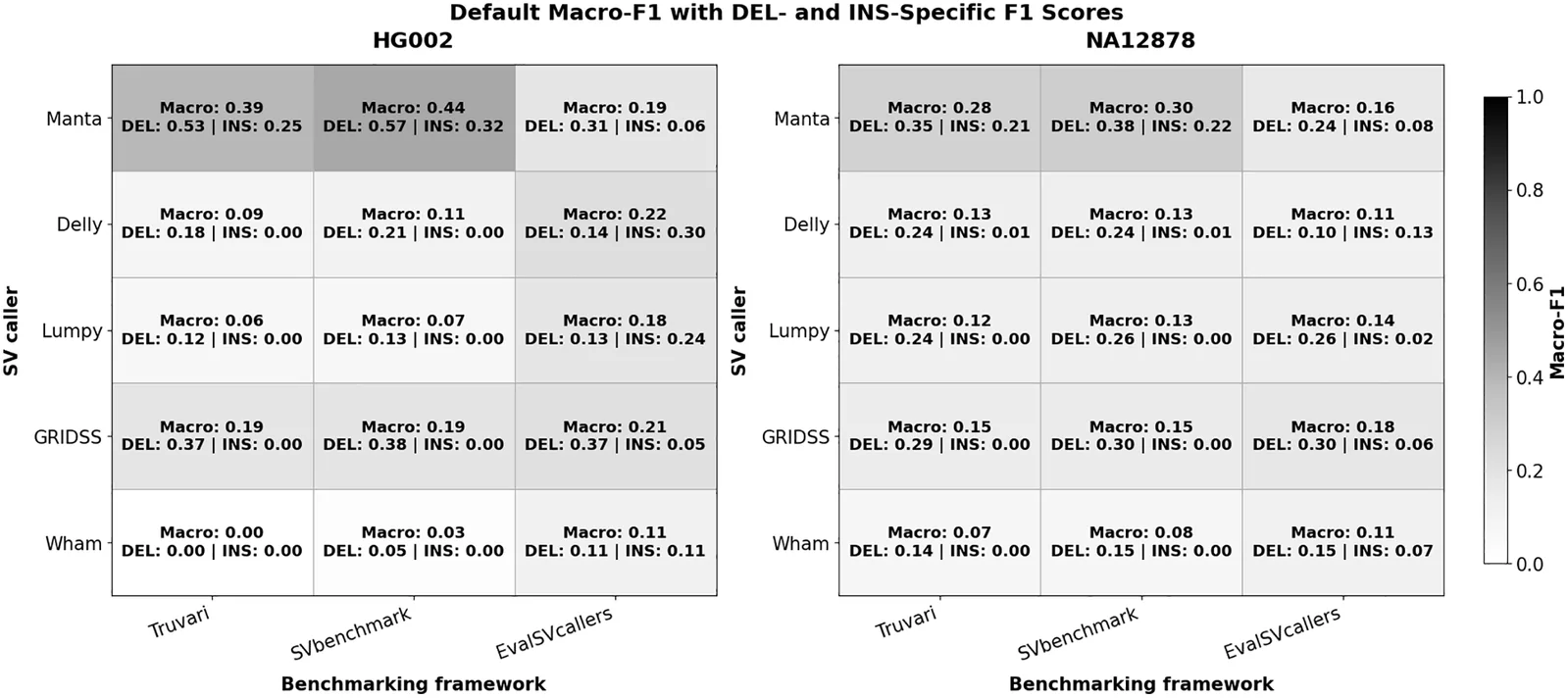
Benchmarking framework effects on type specific SV caller performance.

### 8.4 Baseline TP, FP, and FN characterization

The analysis showed that relatively high precision could coexist with low recall because a comparatively low FP burden could occur simultaneously with a much larger FN burden (Figs A and B in S1 Appendix). This pattern was particularly clear for HG002-Manta. Under Truvari, FP rates for DEL variants were 0.11, 0.12, 0.10, 0.05, and 0.08 across the five size intervals, whereas the corresponding FN rates were 0.69, 0.62, 0.66, 0.24, and 0.47. A similar imbalance was observed for smaller Manta insertions: FP rates were 0.07, 0.07, and 0.18 for the 50–99 bp, 100–499 bp, and 500–999 bp intervals, respectively, while the corresponding FN rates increased from 0.75 to 0.86 and 0.96. SVbenchmark showed the same general pattern, with relatively low Manta FP rates but substantially larger FN rates across several DEL and INS size intervals. Thus, the high-precision/low-recall profile was not explained simply by an excess of false-positive calls; in several strata, the dominant imbalance arose because a substantially larger proportion of truth variants were classified as false negatives. The magnitude and distribution of FP and FN rates nevertheless remained strongly caller- and framework-dependent. For example, HG002 Wham INS variants showed FP rates approaching 1.00 across most size intervals under Truvari and SVbenchmark, whereas EvalSVcallers produced substantially lower FP rates in several of the same size categories (Fig A in S1 Appendix). FN rates for Wham INS remained high under all three frameworks, although their exact values also differed by size and framework (Fig B in S1 Appendix). Overall, the combined FP and FN profiles show that error burdens vary jointly with SV type, SV size, caller, and framework-specific variant-equivalence rules.

### 8.5 Genomic block resampling for default-configuration uncertainty analysis

Genomic block resampling analysis demonstrated that the default benchmarking results were generally stable across the resampled genomic regions. Empirical F1-score ranges were relatively narrow in most sample-caller-framework combinations. Across 200 resampling iterations, Manta consistently achieved the highest average F1-score under both Truvari and SVbenchmark for both HG002 and NA12878 (Fig 7). In contrast, GRIDSS ranked highest in both samples under EvalSVcallers. The narrow empirical F1-score intervals observed in the genomic block-resampling analysis indicate that default benchmarking results were not highly sensitive to the random selection of genomic regions. In other words, caller rankings were generally stable within each benchmarking framework across different genomic subsets. However, the highest-ranking caller changed between frameworks: Manta ranked highest under Truvari and SVbenchmark, whereas GRIDSS ranked highest under EvalSVcallers. This pattern suggests that the observed differences were not primarily driven by regional genomic composition, but by systematic differences in the matching and scoring logic of the benchmarking frameworks. Therefore, benchmarking framework choice itself represents an important source of variation in SV caller evaluation.

**Fig 7:**
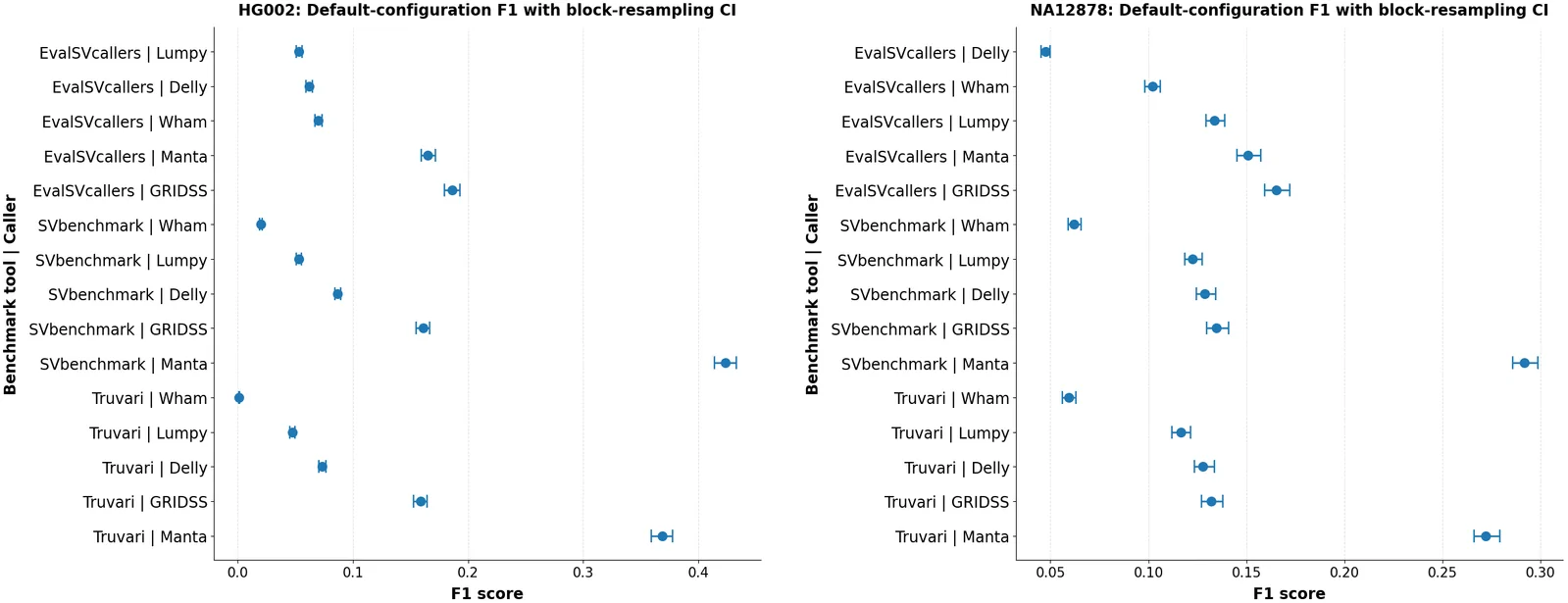
Default configuration F1-score uncertainty estimated using genomic block resampling.

### 8.6 Parameter sensitivity uncertainty analysis

Precision, recall, and F1 scores across all evaluated configurations are shown in Fig 8. Across the evaluated sample-caller-parameter groups, SVbenchmark showed the largest mean parameter-specific F1 range (0.0136), followed by Truvari (0.0086) and EvalSVcallers (0.0053). However, the largest individual parameter-associated deviation was observed with Truvari for NA12878-Lumpy. Increasing *pctseq* from its baseline value of 0.7 to 0.9 reduced F1 from 0.1163 to 0.0455, corresponding to ΔF1 = −0.0708. Across the tested *pctseq* values, F1 decreased progressively from 0.1216 at 0.5 to 0.0455 at 0.9. Similar directional responses to increasing *pctseq* were observed for HG002-Manta and HG002-GRIDSS (Fig 9).

**Fig 8:**
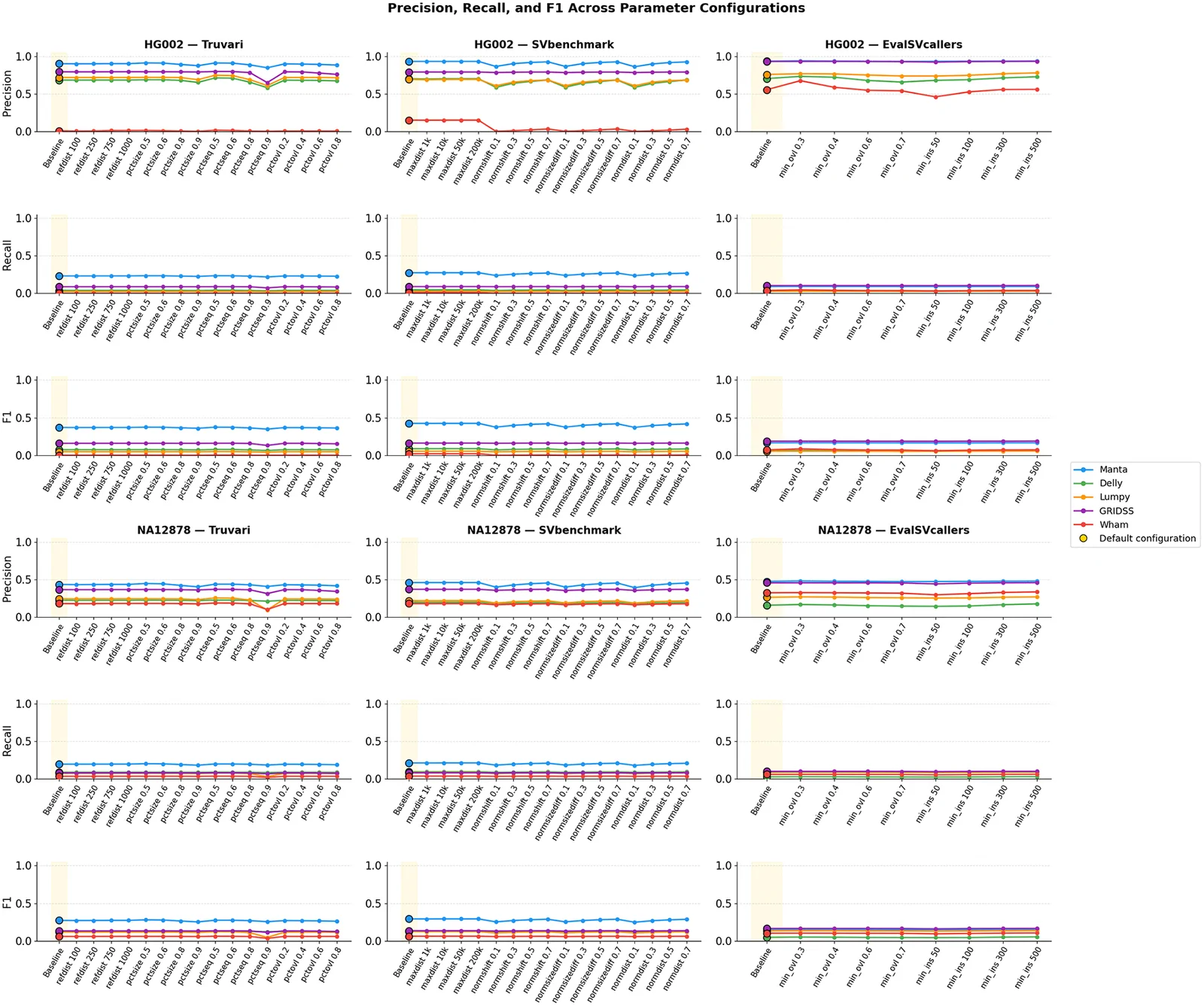
Performance metrics across parameter configurations for HG002 and NA12878.

**Fig 9:**
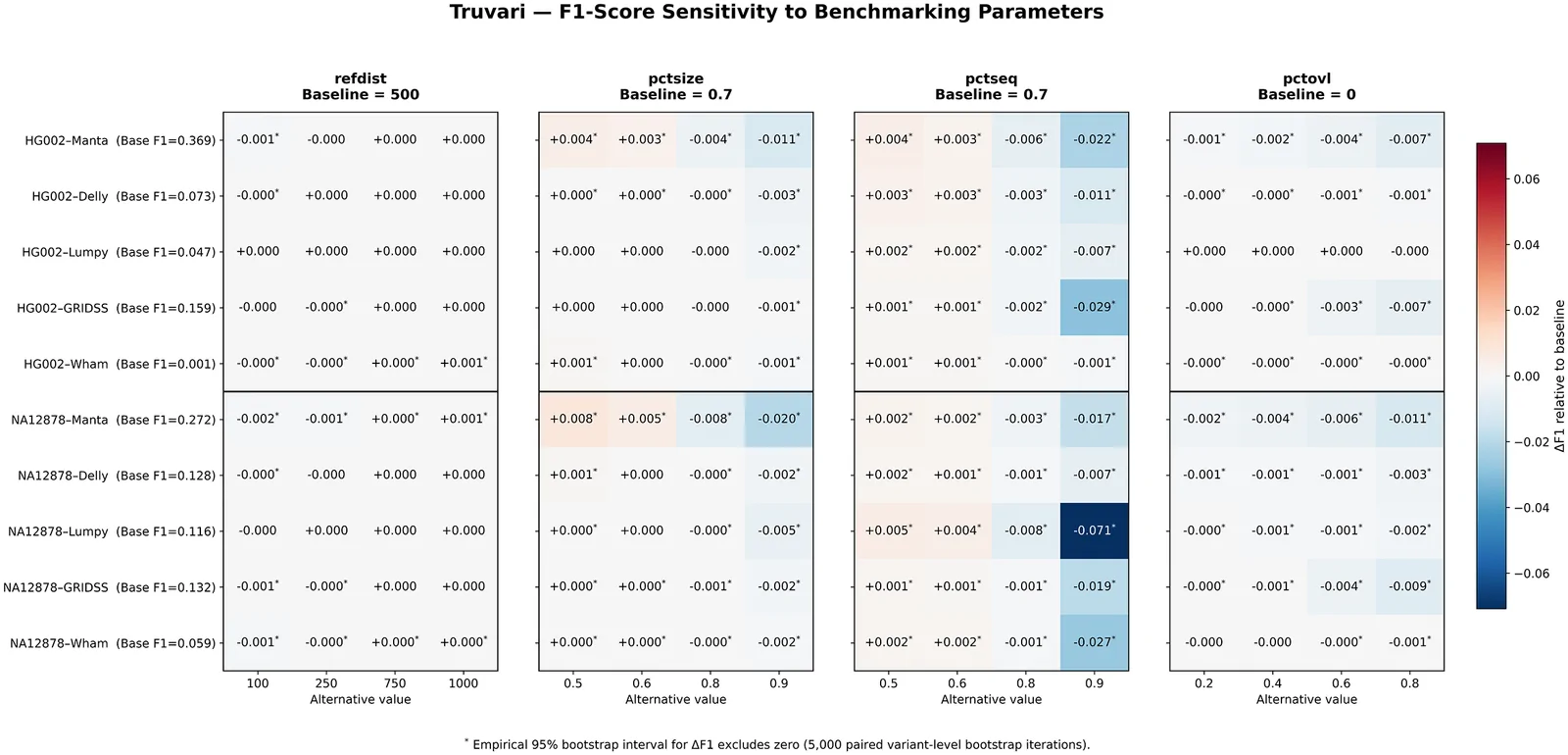
Truvari F1-score sensitivity across tested parameter configurations.

SVbenchmark showed a particularly consistent response for Manta across both samples. In HG002, reducing *normdist*, *normshift*, and *normsizediff* from the baseline value of 1.0 to 0.1 decreased F1 by 0.0538, 0.0507, and 0.0503, respectively. The corresponding decreases in NA12878 were 0.0441, 0.0393, and 0.0388. For example, the HG002–Manta F1-score increased progressively from 0.3700 at *normdist* = 0.1 to 0.3946, 0.4079, and 0.4161 at values of 0.3, 0.5, and 0.7, respectively, approaching the baseline F1-score of 0.4238 at *normdist* = 1.0. A comparable directional pattern was observed for *normshift* and *normsizediff* and was reproduced in NA12878 (Fig 10).

**Fig 10:**
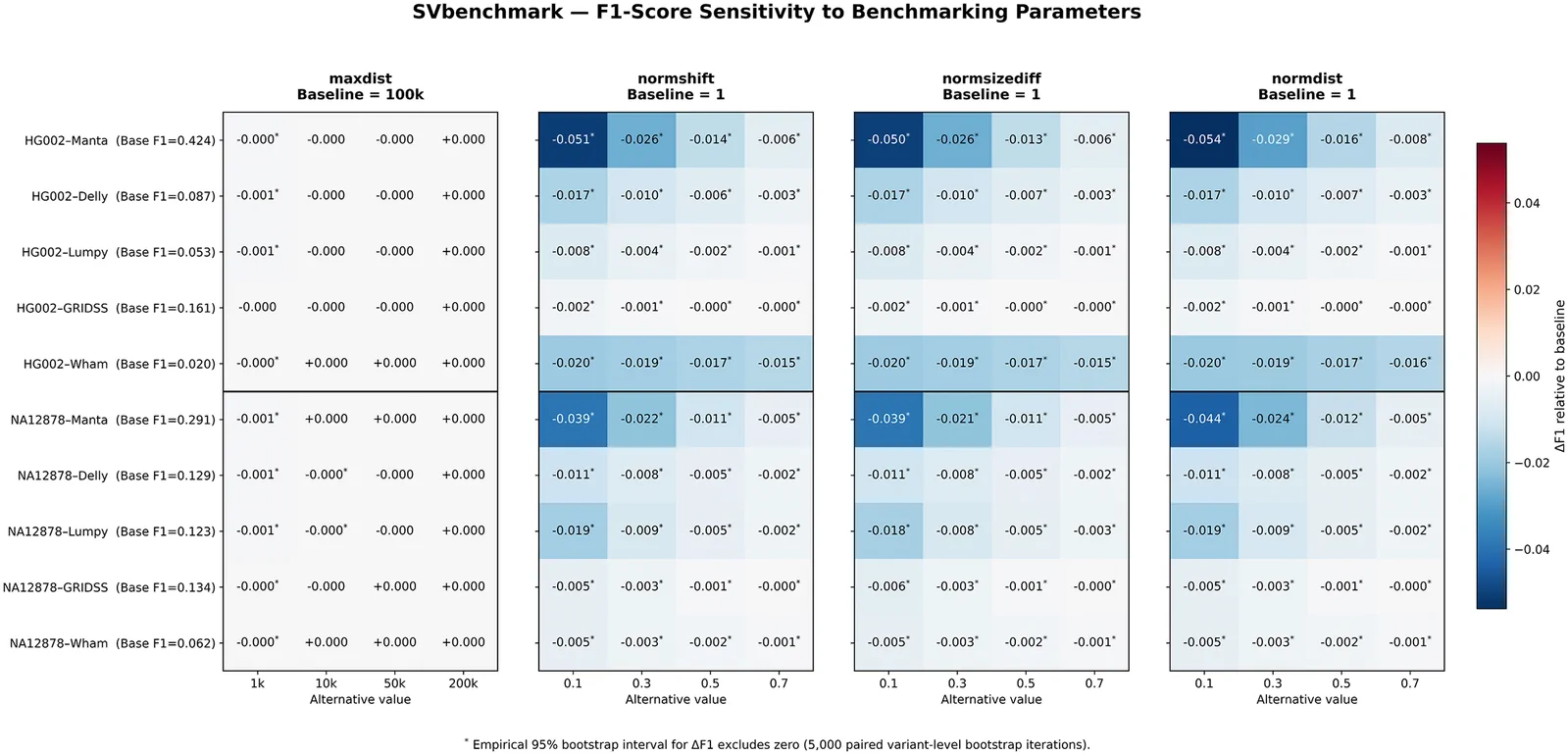
SVbenchmark F1-score sensitivity across tested parameter configurations.

EvalSVcallers showed comparatively smaller overall parameter sensitivity. Its maximum absolute F1-score deviation was 0.0150, observed for HG002-Wham when *min_ovl* was reduced from the baseline value of 0.5 to 0.3. Under this change, F1-score increased from 0.0702 to 0.0852. Increasing *min_ovl* to 0.6 and 0.7 instead produced small decreases to 0.0688 and 0.0681, respectively. The *min_ins* response for the same caller was also modest above the baseline value of 200 bp: F1-score was 0.0702 at *min_ins* = 300 and 0.0713 at *min_ins* = 500, whereas reducing *min_ins* to 50 bp decreased F1-score to 0.0585 (Fig 11).

**Fig 11:**
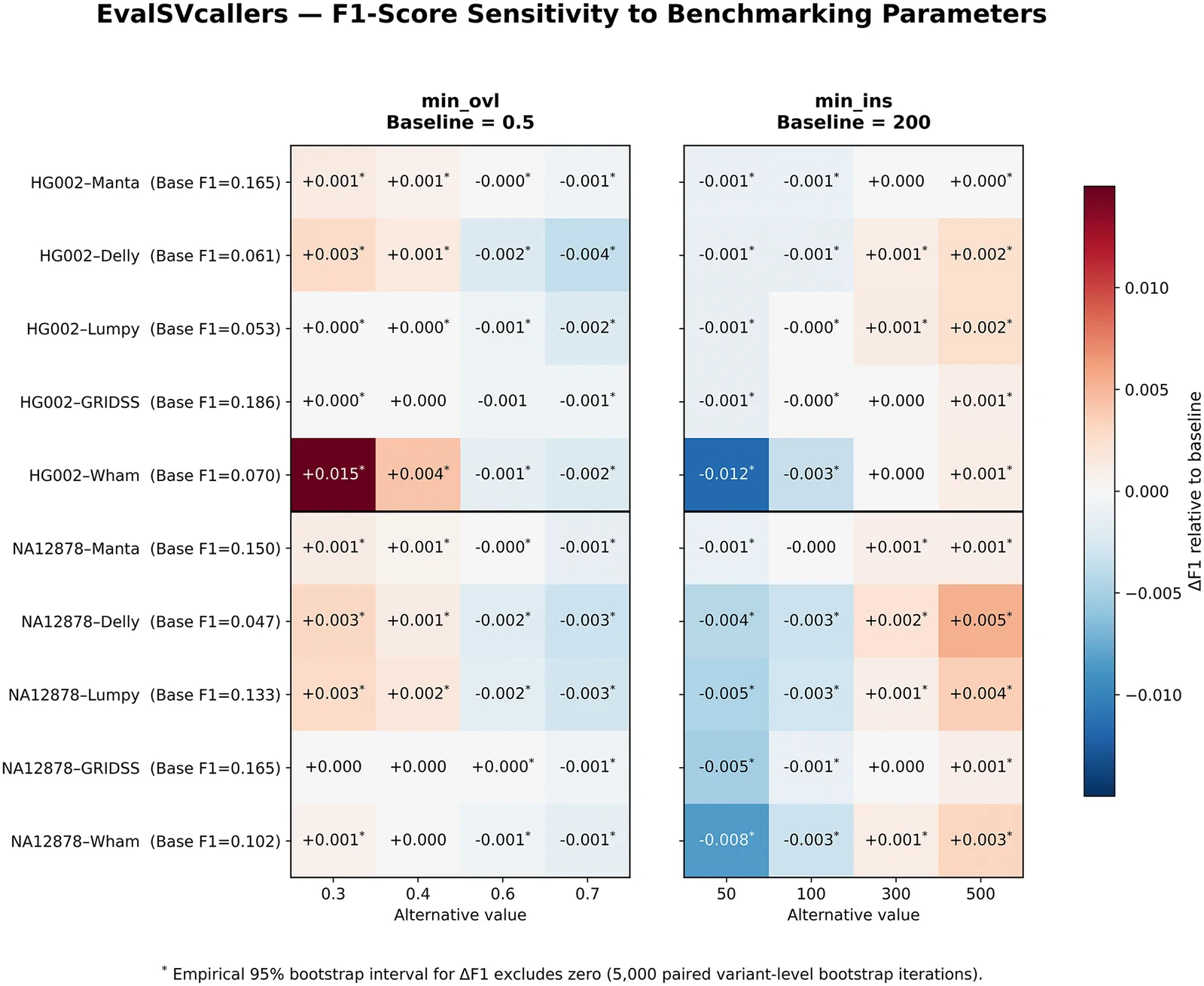
EvalSVcallers F1-score sensitivity across tested parameter configurations.

Overall, the expanded sensitivity analysis demonstrates that the effect of parameterization is not uniform across benchmarking frameworks. SVbenchmark showed the greatest average parameter-associated F1-score variation, whereas Truvari produced the largest individual deviation under the most stringent tested sequence-similarity setting. EvalSVcallers was comparatively stable across the evaluated matching parameters.

### 8.7 Variant level bootstrap analysis

Baseline-relative ΔF1 values and their bootstrap support across the evaluated parameter configurations are shown in Figs 9–11, where an asterisk (*) indicates that the empirical 95% bootstrap interval for ΔF1 obtained from 5,000 paired variant-level bootstrap iterations excluded zero. Among the 400 baseline-alternative comparisons, 323 (80.8%) were bootstrap-supported, including 122 of 160 Truvari comparisons (76.3%), 131 of 160 SVbenchmark comparisons (81.9%), and 70 of 80 EvalSVcallers comparisons (87.5%). The largest parameter effects remained clearly supported. For example, increasing Truvari *pctseq* from 0.7 to 0.9 for NA12878-Lumpy reduced F1 by 0.0708, with a 95% bootstrap interval of [−0.0748, −0.0667] (Fig 9). Similarly, SVbenchmark *normdist* from 1.0 to 0.1 for HG002-Manta reduced F1 by 0.0538 [−0.0566, −0.0511] (Fig 10). For EvalSVcallers, decreasing *min_ovl* from 0.5 to 0.3 for HG002-Wham increased F1 by 0.0154, with a 95% bootstrap interval of [0.0135, 0.0173] (Fig 11). In contrast, changes that were not bootstrap-supported were very small; the largest absolute ΔF1 among these comparisons was only 0.0004. Overall, the major parameter-related F1-score changes remained stable under repeated variant-level resampling, indicating that the observed parameter effects were not primarily driven by random variation in the evaluated variants.

### 8.8 Benchmarking framework concordance across callers and samples

Figs C and D in S1 Appendix present UpSet plots illustrating the overlap of true positive (TP) variant identifications across different benchmarking frameworks under default parameter configuration for each caller and sample (S1 Appendix). Among callers Manta shows the strongest consensus among frameworks in both samples. The three frameworks share 2942 TP variants in NA12878 and 6453 TP variants in HG002. In NA12878, this common intersection represents approximately 97.4% of the Truvari TP set, 90.7% of the SVbenchmark TP set, and 88.5% of the EvalSVcallers TP set. In HG002, it represents approximately 99.5%, 95.9%, and 95.9% of the corresponding TP sets, respectively. These high proportions indicate that most Manta variants classified as TP are interpreted consistently across the three frameworks. The relatively small framework-specific counts (e.g., SVbenchmark = 35 in HG002) indicate that only a limited subset of Manta TP assignments differed across frameworks.

GRIDSS is the caller where EvalSVcallers consistently reports notably more TPs than the other two frameworks in both samples. EvalSVcallers identifies 291 framework-specific TP variants in NA12878 and 426 in HG002. Its total TP set contains 1546 variants in NA12878, compared with 1229 for Truvari and 1259 for SVbenchmark, and 2913 variants in HG002, compared with 2470 and 2488, respectively. Thus, the EvalSVcallers TP set is approximately 1.2 times larger than those produced by the other frameworks in both samples.

Delly shows a complete separation between the TP set identified by EvalSVcallers and those identified by Truvari and SVbenchmark. In HG002, all 1249 EvalSVcallers TP variants are framework-specific, while 1082 variants are shared only between Truvari and SVbenchmark and 147 are unique to SVbenchmark. Similarly, in NA12878, all 1512 EvalSVcallers TP variants are unique to the framework, whereas 1366 TP variants are shared only between Truvari and SVbenchmark, and an additional 107 variants are unique to SVbenchmark. Consequently, no three-framework TP intersection is observed for Delly in either sample. Moreover, the complete Truvari TP set is contained within the SVbenchmark TP set in both samples. This pattern indicates a pronounced framework-specific separation involving EvalSVcallers rather than a difference arising solely from unequal TP set sizes.

In contrast, Lumpy shows substantial three-framework agreement in both samples. The common TP intersection contains 685 variants in HG002 and 1162 variants in NA12878. In HG002, the common intersection corresponds to the complete Truvari TP set and accounts for approximately 91.6% and 87.0% of the SVbenchmark and EvalSVcallers TP sets, respectively. In NA12878, the shared intersection accounts for approximately 99.4% of the Truvari TP set, 88.8% of the SVbenchmark TP set, and 84.5% of the EvalSVcallers TP set.

Wham shows a completely different profile in both figures, and it is most evident in HG002. In HG002, the total number of Wham variants classified as TP decreased from 1062 under EvalSVcallers to 18 under Truvari. This approximately 59-fold difference indicates a pronounced incompatibility between the representation of Wham calls and the matching criteria applied by Truvari in this sample, rather than a biological change in caller performance. Most TP variants identified by EvalSVcallers were not included in the much smaller Truvari TP set, while SVbenchmark produced an intermediate result. A related but less extreme asymmetry is observed in NA12878: EvalSVcallers identifies a total of 934 TP variants. Of these, 367 are unique to EvalSVcallers (39.3%), whereas 533 belong to the three-framework intersection. At the same time, the three-framework intersection accounts for approximately 98.2% of the Truvari TP set, indicating that the smaller Truvari set is almost entirely contained within the larger TP sets.

Figs E and F in S1 Appendix illustrate the pairwise similarity of TP variant sets among three benchmarking frameworks for each SV caller in HG002 and NA12878 (S1 Appendix). The left panels show the Jaccard indices, and the right panels show the overlap coefficient values. The Jaccard index evaluates the intersection of two TP sets relative to their union, while the overlap coefficient normalizes the intersection according to the size of the smaller TP set. Therefore, using both metrics together helps distinguish whether low Jaccard values are due to true TP set divergence or a difference in TP set size. Manta shows the most consistent TP set agreement among the frameworks in both samples. Jaccard values for Manta range from 0.95-0.98 in HG002 and 0.87-0.93 in NA12878. In contrast, the overlap coefficient values being in the range of 0.97-1.00 in both samples indicates that the variant sets classified as TP by the frameworks largely overlap. A similar trend is observed for Lumpy and GRIDSS. Although some Jaccard values are relatively lower in these callers, the overlap coefficient values being close to 1.00 suggests that the low Jaccard values are mostly influenced by the difference in TP set size rather than disagreement between frameworks. Dely shows a significant framework divergence in both samples. While the Jaccard value remains high between Truvari and SVbenchmark, the Jaccard and overlap coefficient values are 0.00 in pairwise comparisons of EvalSVcallers with Truvari and SVbenchmark. This indicates that the variant set classified as TP by EvalSVcallers for Delly does not overlap with the TP sets defined by the other two frameworks. Wham is the caller showing the most significant difference between the two samples. In HG002, the Jaccard values for Wham are very low, and the overlap coefficient values are also low in some comparisons; this indicates a real TP set divergence between frameworks. In contrast, in NA12878, although the Jaccard values for Wham are lower, especially in intersections containing EvalSVcallers, the overlap coefficient values are in the range of 0.98-0.99. This result shows that the low Jaccard values for Wham in NA12878 are largely due to the difference in TP set size, with the smaller TP set largely contained within the larger TP set. Overall, when the overlap coefficient and absolute TP set sizes are considered together, the agreement between frameworks is strong for Manta, Lumpy, and GRIDSS; Delly shows a framework-specific separation involving EvalSVcallers; and Wham shows both TP set divergence and size effects depending on the sample.

Figs G and H in S1 Appendix present Sankey diagrams illustrating how variant outcome labels (TP, FP, and NONE) assigned by one framework are redistributed across outcome categories in other frameworks (S1 Appendix). Each column represents a framework, nodes correspond to outcome categories (TP: green, FP: red, NONE: gray), and link thickness reflects the number of variants following a given transition path. Consistent with the Jaccard patterns in Figs E and F in S1 Appendix, Manta shows the most stable structure in both samples, with a dominant TP → TP → TP flow across all three frameworks. This is in line with its high pairwise Jaccard agreement (0.95–0.98 in HG002 and 0.87–0.93 in NA12878). In HG002, the green TP stream covers nearly the full width of the diagram, whereas in NA12878 the same pattern remains dominant but the FP band is slightly wider. Delly shows the strongest framework disagreement. In both samples, the EvalSVcallers NONE block is visually prominent, and the crossing links indicate extensive label reassignment between Truvari and EvalSVcallers, matching the zero Jaccard overlap previously observed between EvalSVcallers and the other two frameworks. Lumpy and GRIDSS display a more balanced profile, with a strong shared TP stream, a parallel FP → FP → FP band, and only a small NONE component. In both callers, the flows appear cleaner in HG002 than in NA12878. For GRIDSS, the EvalSVcallers TP block is visibly larger than the Truvari TP block in both samples, indicating that EvalSVcallers captures additional TP calls that are largely retained as TP in SVbenchmark as well. Wham shows the most divergent pattern and the clearest sample-specific contrast. In HG002, the Truvari column is dominated by the FP (red) block, with only a very small TP band, directly reflecting the near-zero Jaccard values and the very low F1-score observed for this caller–sample combination. A substantial portion of these Truvari FP calls then shifts into TP in EvalSVcallers and remains TP in SVbenchmark. In NA12878, the picture is more balanced: the Truvari TP band is clearly visible, and although crossing flows remain, the TP → TP → TP path is much more prominent than in HG002. Overall, the Sankey diagrams confirm that Manta showed the greatest cross-framework label consistency in these analyses, whereas Delly and Wham showed substantially greater framework-dependent label redistribution.

### 8.9 Variant level characteristics of cross-framework agreement and disagreement

To move beyond TP set overlap and label transition analyses, the properties of variants showing different levels of cross-framework TP agreement were examined directly. The SV size composition of the cross-framework agreement groups was additionally examined across five size intervals (Table E in S1 Appendix). Size distributions varied across agreement categories for several caller-sample combinations, but no uniform size-dependent pattern of cross-framework disagreement was observed across callers. Variant level characterization of cross-framework TP agreement revealed that disagreement was frequently accompanied by poorer positional and size concordance with the nearest same-type truth variant, although this relationship was not universal (Figs I and J in S1 Appendix). The clearest pattern was observed for DEL variants. For HG002-Manta, variants classified as TP by all three frameworks had a median breakpoint displacement of 0 bp, a median size ratio of 1.00, and a median reciprocal overlap of 1.00. The corresponding values changed to 24 bp, 0.71, and 0.66 for variants classified as TP by two frameworks, and to 163 bp, 0.49, and 0.11 for variants classified as TP by only one framework (Fig I in S1 Appendix). A similar pattern was observed for NA12878-Manta, for which median breakpoint displacement increased from 0 bp to 27 bp and 101 bp across the three agreement categories, while median reciprocal overlap decreased from 1.00 to 0.58 and 0.15, respectively. Lumpy DEL variants showed a comparable reduction in size and interval agreement as cross-framework TP agreement decreased.

However, framework disagreement could not be explained only by breakpoint or size discordance. Delly DEL variants provided a notable counterexample: in HG002, variants classified as TP by two frameworks had a median breakpoint displacement of 9 bp, a median size ratio of 1.00, and a median reciprocal overlap of 0.99, while variants classified as TP by only one framework retained corresponding values of 12 bp, 0.98, and 0.98. In NA12878, both groups showed essentially complete positional, size, and interval agreement with the nearest same-type truth variants. INS variants were also heterogeneous (Fig J in S1 Appendix). In particular, GRIDSS variants classified as TP by only one framework remained closely positioned to the nearest same-type truth variant: the median breakpoint displacement was 2 bp with a median absolute size difference of 1 bp and a median size ratio of 0.98 in HG002, compared with 3 bp, 4 bp, and 0.96, respectively, in NA12878. These results show that breakpoint displacement and size or interval agreement account for an important component of cross-framework disagreement, but a particular discordant classification can arise from representation handling, candidate selection, clustering, one-to-many matching, or another internal framework rule. Representative HG002 loci illustrating both geometrically discordant and geometrically similar framework-specific classifications are provided in Table 1.

**Table 1:** Example representative variants from HG002 unified variant universe illustrating cross-framework agreement and disagreement.

Caller	CHROM	POS	SVTYPE	SVLEN	Truvari	SVbenchmark	EvalSVcallers	Nearest truth POS	Truth SVLEN	Breakpoint displacement (bp)	Size ratio	Reciprocal overlap
Manta	chr1	1598413	DEL	167	TP	TP	TP	1598413	167	0	1.000	1.000
Manta	chr19	4511539	DEL	198	FP	TP	FP	4511365	99	174	0.500	0.000
Delly	chr11	48734742	DEL	2182	NONE	NONE	TP	48734732	2216	10	0.985	0.985
GRIDSS	chr13	77890840	INS	58	FP	FP	TP	77890838	59	2	0.983	N/A

## 9. Discussion

In this study, the outputs of five different SV callers (Manta, Delly, Lumpy, GRIDSS, Wham) on HG002 and NA12878 samples were compared using three different benchmarking frameworks (Truvari, EvalSVcallers, SVbenchmark); parameter sensitivity analyses, genomic block resampling, variant level bootstrap analysis, TP overlap patterns, and label transition flows were examined. The findings show that SV benchmarking reflects not only caller performance but also the matching logic and parameterization of the selected framework.

Genomic block resampling further supported the robustness of the default caller rankings to changes in regional genomic composition. Across 200 resampling iterations, Manta consistently ranked highest under Truvari and SVbenchmark, whereas GRIDSS ranked highest under EvalSVcallers in both samples. These results indicate that the observed framework-specific rankings were not strongly dependent on the particular subset of genomic regions included in an iteration. Thus, within each framework, caller rankings were relatively robust to regional genomic composition, whereas the identity of the highest-ranking caller differed across frameworks according to how variant equivalence was defined.

The additional TP/FP/FN characterization helps explain why relatively high precision can coexist with low recall in several caller–framework combinations. High precision indicates that a substantial proportion of the caller records admitted to the evaluation could be associated with truth variants, but it does not imply that the caller covered a large fraction of the truth set. In several cases, particularly Manta, the principal limitation was instead the large number of truth variants remaining unmatched. This imbalance was stronger for INS than DEL in both samples, and the size-stratified analysis further showed that both caller-side FP rates and truth-side FN rates varied substantially across SV length intervals, with the imbalance between the two depending on caller, framework, and SV type (Figs A and B in S1 Appendix). Nevertheless, these patterns should not be interpreted solely as intrinsic caller detection failures. The same caller output could produce markedly different matched and unmatched distributions across frameworks, as illustrated by Wham and GRIDSS.

Cross-framework TP agreement varied substantially across callers and samples. UpSet, Jaccard indices, and overlap coefficient analyses collectively support this interpretation (Figs C-F in S1 Appendix). While the Jaccard index more rigorously measures pairwise similarity between TP sets by penalizing differences in total set size, the overlap coefficient helps assess whether one framework’s TP set largely overlaps with another framework’s TP set. This distinction is important because low Jaccard values do not always imply true disagreement between frameworks. For Manta, high Jaccard values and overlap coefficients close to 1.00 indicated strong agreement between frameworks in both samples. For Lumpy and GRIDSS, some lower Jaccard values accompanied by high overlap coefficients suggest that the apparent mismatch in most cases stems from differences in TP set sizes rather than a complete divergence of TP calls. In contrast, pairwise comparisons involving EvalSVcallers in Delly showed both low Jaccard and low overlap coefficient values, indicating stronger framework-specific TP-set divergence. Wham showed the most prominent sample-dependent behavior: in HG002, low Jaccard and low overlap coefficient values indicated true TP set separation, while in NA12878, low Jaccard but high overlap coefficient values suggested that the smaller TP set was largely contained within the larger TP set. These patterns show that inter-framework mismatches can stem from differences in true variant matching decisions or unequal TP set sizes, and that both Jaccard index and overlap coefficient values should be evaluated together to distinguish between these two situations.

The parameter sensitivity analysis demonstrated that the magnitude of parameter-associated variation varied across benchmarking frameworks, callers, samples, and individual matching criteria (Figs 9–11). Across the evaluated sample-caller-parameter groups, SVbenchmark showed the largest mean parameter-specific F1-score range (0.0136), followed by Truvari (0.0086) and EvalSVcallers (0.0053). However, the largest individual deviation occurred under Truvari for NA12878-Lumpy, where increasing *pctseq* from 0.7 to 0.9 reduced F1-score by 0.0708. Thus, a lower framework level average sensitivity does not preclude substantial responses to particular parameter settings for individual caller-sample combinations. SVbenchmark showed a particularly consistent response for Manta when the normalized matching criteria were varied. Lower values of *normdist*, *normshift*, and *normsizediff* were associated with progressively lower F1-scores in both HG002 and NA12878, with the largest decreases occurring at 0.1. EvalSVcallers, in contrast, showed comparatively smaller changes across the tested *min_ovl* and *min_ins* values, with a maximum absolute F1-score deviation of 0.0150.

The paired variant level bootstrap analysis further supported the robustness of the parameter-associated F1-score changes. Empirical 95% bootstrap intervals excluded zero for 323 of the 400 baseline-alternative comparisons (80.8%), including 76.3% of Truvari, 81.9% of SVbenchmark, and 87.5% of EvalSVcallers comparisons. Importantly, the largest observed parameter effects remained clearly supported under repeated resampling, whereas changes without bootstrap support were negligible, with a maximum absolute ΔF1 of only 0.0004. These findings indicate that the major parameter-associated effects were not primarily driven by random variation in the evaluated variants.

Sankey diagrams show that label reassignments across frameworks were asymmetric, with variants classified as TP in one framework being reassigned as FP or NONE in another (Figs G and H in S1 Appendix). The additional variant level characterization provides further insight into the properties associated with these disagreements (Figs I and J in S1 Appendix). For several DEL caller-sample combinations, particularly Manta and Lumpy, decreasing cross-framework TP agreement was accompanied by greater breakpoint displacement, poorer size agreement, and reduced reciprocal overlap. For HG002-Manta, for example, median breakpoint displacement increased from 0 bp among variants classified as TP by all three frameworks to 163 bp among variants classified as TP by only one framework, while median reciprocal overlap decreased from 1.00 to 0.11. However, this relationship was not universal. Delly DEL variants retained very high positional, size, and interval agreement with the nearest same-type truth variants even when their TP classifications varied across frameworks, while framework-specific GRIDSS INS variants also remained closely aligned in position and size.

Sample-specific metric values should be interpreted in light of differences in genomic evaluation scope. HG002 was evaluated within GIAB high-confidence benchmark regions, whereas no equivalent region restriction was available for NA12878. Restricting evaluation to high-confidence regions can affect precision, recall, and F1 by excluding genomic regions in which variant detection and matching are more uncertain. Higher precision or F1-score in one sample does not necessarily indicate intrinsically better caller performance, because the reported values also reflect truth set scope, variant representation, and the genomic regions included in the evaluation. Wham’s differing benchmarking outcomes between the two samples further illustrate this context dependence.

The present analysis was intentionally restricted to DEL and INS variants, consistent with the SV classes represented in the truth sets used in this study, thereby providing a controlled setting in which framework-specific matching behavior could be examined without the additional representational complexity introduced by more complex SV classes. Consequently, the observed framework-dependent differences should not be assumed to generalize quantitatively to complex rearrangements, repetitive regions, or low-mappability regions. In these more challenging contexts, breakpoint ambiguity, alternative representations of the same event, reduced mapping confidence, and one-to-many variant representations may further increase disagreement between benchmarking frameworks. Evaluation of these contexts using dedicated difficult-region and complex-variant benchmark resources remains an important direction for future work. Likewise, benchmarking resources such as the Tandem Repeat and CMRG truth sets, as well as T2T-based genome representations may support more targeted evaluation in difficult genomic contexts that are not fully captured by genome-wide benchmark designs.

Taken together, these findings underscore that a universally accepted definition of an “equivalent variant” remains absent in SV benchmarking. Precision, recall, and F1-scores should therefore not be interpreted as absolute measures of caller accuracy; rather, they are conditional summary statistics determined by the assumptions, parameters, truth set scope, and matching logic of the selected benchmarking framework. Researchers should consequently select evaluation frameworks according to their study objectives, the variant classes of interest, the available high-confidence truth resources, and the desired balance between permissive and stringent variant matching. Transparent reporting of the benchmarking framework, parameter settings, preprocessing steps, and evaluated genomic scope is essential for reproducibility and cross-study comparison. From a practical perspective, SV benchmarking should be treated as a study-design decision rather than a purely technical post-processing step. When biological conclusions depend on relatively small differences between callers, evaluation under more than one benchmarking framework can help determine whether the observed ranking is framework-dependent. Parameters that directly control variant equivalence should also be examined over a reasonable range when they affect the conclusions, and absolute metric changes should be considered alongside relative changes, particularly when baseline F1-scores are low. Cross-framework TP-set comparisons can further help distinguish broad agreement from framework-specific classification differences that may not be apparent from aggregate F1-scores alone. Default parameters may provide a useful starting point, but they should not automatically be assumed to represent the most appropriate evaluation setting for every caller-sample combination.

## 10. Conclusions

This study shows that SV benchmarking results can vary substantially depending on the selected framework and parameter settings. Even when the same query and truth sets are evaluated, differences in variant equivalence definitions, matching criteria, and decision thresholds can lead to different variant classifications and performance metrics. Precision, recall, and F1-scores should therefore be interpreted as framework-dependent summary measures rather than absolute measures of SV caller accuracy.

Default configuration and genomic block resampling analyses showed that caller rankings were relatively robust within individual frameworks but differed across frameworks. The expanded parameter sensitivity analysis further demonstrated that sensitivity was not uniform across frameworks or caller-sample combinations. SVbenchmark showed the greatest mean parameter specific F1-score variation, whereas Truvari produced the largest individual deviation under the most stringent tested *pctseq* setting. EvalSVcallers showed comparatively smaller variation across the evaluated *min_ovl* and *min_ins* values. Paired variant level bootstrap resampling showed that most parameter associated F1-score changes were robust to variation in the evaluated variants, with empirical 95% intervals excluding zero in 323 of 400 baseline-alternative comparisons (80.8%). UpSet plots, Jaccard indices, overlap coefficients, and Sankey diagrams further showed that cross-framework disagreement can reflect both differences in TP set size and differences in variant classification across frameworks. Cross-framework variant level characterization further showed that discordant TP assignments were often associated with greater breakpoint displacement and poorer size or interval agreement, particularly for DEL variants. However, framework specific classifications also persisted for variants that remained closely aligned to same-type truth variants in position and size, demonstrating that geometric disagreement alone does not fully explain cross-framework variation. Additional baseline variant level characterization showed that the frequent high-precision/low-recall pattern was primarily associated with a large burden of FN rather than uniformly high numbers of FP. This imbalance was generally more pronounced for INS than DEL and varied across SV-size strata, while substantial framework specific differences indicated that TP, FP, and FN profiles reflect the interaction between caller output characteristics and framework specific matching logic.

Consequently, the absence of both a universally accepted definition of SV equivalence and a single standard benchmarking framework requires transparent reporting of evaluation logic, parameter settings, preprocessing procedures, truth set scope, and genomic evaluation domain. Reliable and reproducible SV benchmarking should therefore be designed according to the study objective, the variant classes of interest, and the available benchmark resources rather than assuming that any single framework provides an absolute measure of accuracy.

### Key Points

- SV benchmarking results depend on both the selected evaluation framework and its parameterization. Systematic variation of breakpoint distance, size similarity, sequence similarity, overlap, and normalized matching criteria produced caller- and sample-dependent changes in the reported metrics.
- Parameter sensitivity was not uniform across benchmarking frameworks. SVbenchmark showed the largest mean parameter-specific F1 variation, Truvari produced the largest individual F1 deviation under the most stringent tested *pctseq* setting, and EvalSVcallers showed comparatively smaller variation across the evaluated matching parameters. Paired variant level bootstrap resampling supported the robustness of most parameter associated F1-score changes: empirical 95% intervals excluded zero in 323 of 400 baseline-alternative comparisons (80.8%), while unsupported changes were limited to very small absolute F1-score differences.
- Genomic block resampling showed that default caller rankings were relatively stable within frameworks but could vary across frameworks. Manta ranked highest under Truvari and SVbenchmark, whereas GRIDSS ranked highest under EvalSVcallers in both evaluated samples. Whereas genomic block resampling assessed robustness to changes in regional genomic composition, variant level bootstrap resampling specifically evaluated the stability of parameter associated metric changes to variation in the evaluated variant sets.
- Variant level characterization showed that high precision could coexist with low recall because many truth variants remained unmatched even when a large proportion of evaluated caller records were successfully matched. The FN was generally greater for INS than DEL and varied across SV-size strata, while its magnitude remained framework dependent.
- Jaccard indices and overlap coefficients provide complementary information about TP-set agreement. A low Jaccard index can result from unequal TP-set sizes even when the smaller set is largely contained within the larger set, whereas simultaneously low Jaccard and overlap coefficients indicate stronger TP-set separation.
- Cross-framework variant level characterization showed that reduced TP agreement was often accompanied by greater breakpoint displacement and poorer size or interval agreement, particularly for DEL variants. However, Delly DEL and GRIDSS INS examples showed that framework specific TP assignments can persist despite close positional and size agreement with same-type truth variants.
- Precision, recall, and F1 scores, as well as the resulting TP, FP, and FN classifications, should be interpreted as conditional on the benchmarking framework and its matching assumptions rather than as absolute measures of SV caller accuracy. Transparent reporting of matching logic, parameter settings, truth set scope, preprocessing, and genomic evaluation domain is essential for reproducible and comparable SV benchmarking.

## Supporting information

S1 Appendix Algorithmic logic of SV benchmarking frameworks. Detailed worked examples illustrating the sequential matching logic and acceptance criteria applied by Truvari, EvalSVcallers, and SVbenchmark to the same truth and query variants. Table A. Truth variants and associated variant characteristics. Truth set variants used in the conceptual comparison of the three benchmarking frameworks, including chromosome, genomic position, variant type, SV length, genotype, and haplotype presence. Table B. Query variants and associated variant characteristics. Query variants used in the conceptual comparison of the three benchmarking frameworks, including chromosome, genomic position, variant type, SV length, genotype/phasing information, and haplotype presence. Table C. Command-level reproducibility workflow and repository components. Summary of the computational workflow and repository components supporting reproducibility, including preprocessing, SV calling, baseline benchmarking, parameter-sensitivity analysis, genomic block resampling, variant level bootstrap analysis, cross-framework characterization, figure generation, software environments, and repository validation. Table D. Variant counts across preprocessing steps. Caller-specific record counts for HG002 and NA12878 before and after OctopuSV representation standardization and subsequent preprocessing steps, including BND resolution, DEL/INS selection, autosomal restriction, minimum-size filtering, and the final HG002 high-confidence BED restriction where applicable. Table E. SV-size distribution according to cross-framework TP agreement. Distribution of DEL and INS query variants across five SV-size intervals according to whether they were classified as TP by all three frameworks, exactly two frameworks, or only one framework, shown separately for each sample and caller. Fig A. Caller-side baseline FP burden by SV type and size. Caller-side false-positive burden under the baseline configurations of Truvari, EvalSVcallers, and SVbenchmark, stratified by caller, sample, SV type, and SV-size interval. Fig B. Truth-side baseline FN burden by SV type and size. Truth-side false-negative burden under the baseline configurations of Truvari, EvalSVcallers, and SVbenchmark, stratified by caller, sample, SV type, and SV-size interval. Fig C. Overlap of TP variants across frameworks for HG002. UpSet plots showing the overlap of variants classified as true positive by Truvari, EvalSVcallers, and SVbenchmark under their default configurations for each SV caller in HG002. Fig D. Overlap of TP variants across frameworks for NA12878. UpSet plots showing the overlap of variants classified as true positive by Truvari, EvalSVcallers, and SVbenchmark under their default configurations for each SV caller in NA12878. Fig E. Pairwise Jaccard index and overlap coefficient of TP variants across frameworks for HG002. Pairwise comparison of framework-specific TP variant sets in HG002 using the Jaccard index and overlap coefficient. Together, these measures distinguish TP set divergence from differences primarily caused by unequal TP set sizes. Fig F. Pairwise Jaccard index and overlap coefficient of TP variants across frameworks for NA12878. Pairwise comparison of framework-specific TP variant sets in NA12878 using the Jaccard index and overlap coefficient. Together, these measures distinguish TP set divergence from differences primarily caused by unequal TP set sizes. Fig G. Sankey diagram showing the flow of labels (TP, FP, NONE) across frameworks for HG002. Cross-framework transitions of query-variant outcome labels among Truvari, EvalSVcallers, and SVbenchmark for each caller in HG002. NONE indicates the absence of a reported framework outcome and is used only as a cross-framework bookkeeping category. Fig H. Sankey diagram showing the flow of labels (TP, FP, NONE) across frameworks for NA12878. Cross-framework transitions of query-variant outcome labels among Truvari, EvalSVcallers, and SVbenchmark for each caller in NA12878. NONE indicates the absence of a reported framework outcome and is used only as a cross-framework bookkeeping category. Fig I. DEL characteristics by cross-framework TP agreement. Breakpoint displacement, size ratio, and reciprocal overlap of DEL variants classified as TP by all three frameworks, exactly two frameworks, or only one framework, shown separately across caller-sample combinations. Fig J. INS characteristics by cross-framework TP agreement. Breakpoint displacement, absolute size difference, and size ratio of INS variants classified as TP by all three frameworks, exactly two frameworks, or only one framework, shown separately across caller-sample combinations.
(DOCX)
