# Supplementary material for: Structural Variant benchmarking frameworks: Parameterization, matching logic, and evaluation assumptions presented through HG002 and NA12878
Source: PLoS Comput Biol. 2026 Sep 23;22(9):e1014824. doi: 10.1371/journal.pcbi.1014824 (PMC13623092; doi:10.1371/journal.pcbi.1014824)
Supplement: Supplementary file 1 [file pcbi.1014824.s001.docx]

**Supplementary Material**

**for**

**Structural Variant Benchmarking Frameworks:**

**Parameterization, Matching Logic, and Evaluation Assumptions Presented Through HG002 and NA12878**

Gamze Maden, Mehmet Baysan, Nizamettin Aydın

### Contents

Contents......................................................................................................................................1

S1 Appendix. Algorithmic Logic of SV Benchmarking Frameworks.......................................3

Table A: Truth variants and associated variant characteristics..................................................3

Table B: Query variants and associated variant characteristics.................................................3

Truvari (bench)...........................................................................................................................4

Equation (1): Breakpoint distance criterion...............................................................................4

Equation (2): Size similarity evaluation.....................................................................................4

Equation (3): Reciprocal overlap and genomic span..................................................................4

Equation (4): Reciprocal overlap and genomic span..................................................................5

Equation (5-6): Evaluation window definition...........................................................................5

Equation (7-8): Alternate haplotype reconstruction...................................................................5

Equation (9-11): Edit distance and sequence similarity.............................................................6

EvalSVcallers ............................................................................................................................6

Equation (12-13): Fixed breakpoint proximity filtering.............................................................7

Equation (14): Overlap length computation...............................................................................7

Equation (15-16): Reciprocal overlap conditions......................................................................7

SVbenchmark ............................................................................................................................8

Equation (17): Candidate-pair gating proximity........................................................................8

Equation (18-20): Maximum shift and normalized distance......................................................9

Equation (21): Normalized size difference..............................................................................10

Equation (22): Normalized edit distance..................................................................................10

Command-Level Reproducibility Workflow...........................................................................10

Table C: Command-level reproducibility workflow and repository components....................10

Table D: Variant counts across preprocessing steps................................................................11

Table E: SV-size distribution according to cross-framework TP agreement...........................12

Fig A: Caller-side baseline FP burden by SV type and size....................................................16

Fig B: Truth-side baseline FN burden by SV type and size.....................................................17

Fig C:Overlap of TP variants across frameworks for HG002..................................................18 Fig D: Overlap of TP variants across frameworks for NA12878.............................................19 Fig E: Pairwise Jaccard index and overlap coefficient.............................................................20

Fig F: Pairwise Jaccard index and overlap coefficient...............................................................21

Fig G: Sankey diagram.............................................................................................................22 Fig H: Sankey diagram.............................................................................................................23
Fig I: DEL characteristics by cross-framework TP agreement................................................24
Fig J: INS characteristics by cross-framework TP agreement.................................................24

References................................................................................................................................25

**S1** **Appendix.** **Algorithmic Logic of SV Benchmarking Frameworks**

**Table A.** Truth variants and associated variant characteristics.

| **Variant ID** | **Chromosome** | **POS** | **END** | **ALT** | **Type** | **SVLEN** | **Genotype** | **Haplotype Presence** |
| --- | --- | --- | --- | --- | --- | --- | --- | --- |
| Truth-DEL | chr2 | 130061662 | 130062293 | C | DEL | -631 | 1\|1 | Homozygous alternate; present on both haplotypes |
| Truth-SNP | chr2 | 130062520 | 130062520 | C | SNP (T→C) | – | 1\|1 | Homozygous alternate; present on both haplotypes |
| Truth-indel | chr2 | 130062768 | 130062768 | A | Small indel (AG→A) | – | 1\|1 | Homozygous alternate; present on both haplotypes |

**Table B.** Query variants and associated variant characteristics.

| **Variant ID** | | **Chromosome** | **POS** | **END** | **ALT** | **Type** | **SVLEN** | **Genotype / Phasing** | **Haplotype Presence** |
| --- | --- | --- | --- | --- | --- | --- | --- | --- | --- |
|  | Query-DEL | chr2 | 130061662 | 130062293 | C | DEL | −631 | 0/1, PGT=0\|1 | Present on one haplotype only |
|  | Query-INS-1 | chr2 | 130062308 | 130062308 | A | INS | 539 | 0/1, PGT=1\|0 | Present on the opposite haplotype |
|  | Query-INS-2 | chr2 | 130062642 | 130062642 | A | INS | 813 | 0/1, PGT=1\|0 | Present on the same haplotype as INS-1 |

**Truvari (bench)**

Truvari (English et al., 2022; https://github.com/acenglish/truvari) compares two VCF files to identify matching SV pairs based on configurable similarity criteria. For the deletion (Truth-DEL and Query-DEL) variants, compatibility is first assessed using the breakpoint distance criterion defined by the *--refdist* parameter (default: 500 bp). The truth variant breakpoint interval is first expanded by the specified breakpoint tolerance and the maximum of the adjusted start positions (POS) and the minimum of the adjusted end positions (END) from the truth and query variants are determined. If the maximum adjusted POS is smaller than the minimum adjusted END, the breakpoint distance criterion is satisfied and the variants are considered compatible as defined in Equation (1).

$max\left( {truth}_{POS} - refdist, {query}_{POS} \right)<min\left( {truth}_{END} + refdist, {query}_{END} \right)$	(1)

$$max\left( 130061662 - 500, 130061662 \right)<min\left( 130062293 + 500, 130062293 \right) ✓$$

Following positional compatibility, size similarity is evaluated using the *--pctsize* threshold (default: 0.7). The absolute SVLEN values of the truth and query variants are both 631 bp. The size similarity is calculated as the ratio of the smaller absolute SV length to the larger absolute SV length as defined in Equation (2).

$L_{truth}$= length of truth variant

$L_{query}$= length of query variant

$$size similarity=\frac{\left( \left| L_{truth} \right|,\left| L_{query} \right| \right)}{\left( \left| L_{truth} \right|,\left| L_{query} \right| \right)} (2)$$

$$=\frac{\left( 631, 631 \right)}{\left( 631, 631 \right)}=\frac{\left( 631 \right)}{\left( 631 \right)}=1.0\geq0.7✓$$

Following the breakpoint distance and size similarity checks, reciprocal overlap is evaluated using the *--pctovl* parameter (default: 0.0). To determine whether the truth and query variants share an overlapping genomic interval, the maximum of the start coordinates (POS) and the minimum of the end coordinates (END) are determined. If the maximum POS is smaller than the minimum END, the two variants are considered to have an overlapping genomic interval (Equation (3)).

$max\left( {truth}_{POS}, {query}_{POS} \right)<min\left( {truth}_{END},{query}_{END} \right)$	(3)

$$max\left( 130061662, 130061662 \right)<min\left( 130062293, 130062293 \right) ✓$$

The overlap length is calculated as the difference between the minimum of the two end coordinates and the maximum of the two start coordinates. This value represents the number of overlapping bases. The reciprocal overlap is then calculated by dividing the overlap length by the larger of the two variant interval lengths (Equation (4)).

$$reciprocal overlap=\frac{\left( {truth}_{END}, {query}_{END} \right) -\left( {truth}_{POS}, {query}_{POS} \right)}{\left( {truth}_{END}-{truth}_{POS}, {query}_{END}-{query}_{POS} \right)} (4)$$

$$= \frac{min\left( 130062293, 130062293 \right) -max\left( 130061662, 130061662 \right)}{max\left( 130062293 - 130061662, 130062293 - 130061662 \right)}=\frac{631}{631} > 0.0 ✓$$

Finally, sequence similarity is evaluated using the *--pctseq* parameter (default: 0.7). An evaluation window spanning both truth and query variants is defined using their start (POS) and end (END) coordinates as shown in Equation (5) and (6).

start = min(${truth}_{POS}$, ${query}_{POS}$)	(5)

= $min\left( 130061662, 130061662 \right)=130061662$

end = max(${truth}_{END}$, ${query}_{END}$)	(6)

=$max\left( 130062293, 130062293 \right)=130062293$

Alternate haplotype sequences are constructed by inserting the variant alleles into the reference sequence. A_1_ and A_2_ are the alternate alleles of the truth and query variants, respectively and A_1_ and A_2_ have identical sequences in this example “C”. H_1_ and $H_{2}$represent the reconstructed haplotypes implied by each variant as shown in Equations (7) and (8).

H_1_ = ref[start : ${truth}_{POS}$] + A_1_ + ref[${truth}_{END}$ : end]	(7)

= ref[$130061662:130061662]$ + “C” + ref[$130062293: 130062293]$ = C

H_2_ = ref[start : ${query}_{POS}$] + A_2_ + ref[${query}_{END}$ : end]	(8)

= ref[$130061662:130061662]$ + “C” + ref[$130062293: 130062293]$ = C

Edit distance counts the minimum number of base-level operations (insertions, deletions, substitutions) needed to transform one haplotype sequence into the other. Since H_1_ and H_2_ are identical, no edits are required; therefore, edit distance is 0 (Equation (9)).

edit distance($H_{1}$, $H_{2}$)=0	(9)

$\left| H_{1} \right|$ and $\left| H_{2} \right|$ denote sequence lengths of haplotype-1 and haplotype-2. Each haplotype sequence consists of a single base “C”; therefore, both sequences have a length of 1, giving a total sequence length of 2 (Equation (10)).

$L_{total}=\left| H_{1} \right|+\left| H_{2} \right|$	(10)

$$L_{total}=1+1=2$$

Sequence similarity is defined as one minus the normalized edit distance. Since the edit distance is 0 and the total sequence length is 2, the resulting sequence similarity is 1.0, which satisfies the threshold of 0.7 (Equation (11)).

$$sequence similarity = 1-\frac{edit distance\left( H_{1}, H_{2} \right)}{\left| H_{1} \right|+ \left| H_{2} \right|} (11)$$

$$=1-\frac{0}{1+1}=1.0\geq0.7 ✓$$

As the truth and query deletion variants meet all positional, size-based, overlap, and sequence similarity thresholds, the deletion call in the query set is classified as a true positive (TP). In contrast, since no insertion (INS) events are present in the truth set, all insertion calls in the query callset are classified as false positives (FPs), while truth variants of the evaluated structural variant types that do not have a corresponding match in the query callset are classified as false negatives (FNs).

**EvalSVcallers**

EvalSVcallers (Kosugi et al., 2019; https://github.com/stat-lab/EvalSVcallers) evaluates SV caller performance based on reciprocal overlap against a reference set. For the deletion (Truth-DEL and Query-DEL) variants, evaluation under the EvalSVcallers framework begins with chromosome and SV type concordance. Both the truth and query deletion variants are located on the same chromosome (chr2) therefore the query variant is considered as a candidate.

Candidate variant pairs are then filtered using a fixed breakpoint proximity threshold that is hard-coded in EvalSVcallers (var_sd = 125 bp). This step requires both start and end coordinates of the query and truth variants to fall within this tolerance (Equation (12)(13)). Variants passing this preliminary filtering step are then evaluated using reciprocal overlap criteria to determine true positive matches.

∣${truth}_{POS}$−${query}_{POS}$∣ ≤ 125 bp	(12)

∣$130061662$−$130061662$∣ ≤ 125 bp $✓$

∣${truth}_{END}$−${query}_{END}$∣ ≤ 125 bp	(13)

∣$130062293$−$130062293$∣ ≤ 125 bp $✓$

Following positional filtering, reciprocal overlap is assessed using the *--min_ovl* parameter (default: 0.5). The overlap length corresponds to the genomic interval shared by the reference and query variants, computed as the difference between the minimum end coordinate and the maximum start coordinate as defined in Equation (14). Since both variants have identical breakpoints, the overlap equals the full variant length (631 bp).

$overlap=min\left( {truth}_{END}, {query}_{END} \right) -max\left( {truth}_{POS}, {query}_{POS} \right)$	(14)

$overlap=min \left( 130062293, 130062293 \right)-max \left( 130061662, 130061662 \right)$= 631 bp

The overlap satisfies the reciprocal overlap condition, as the shared region (631 bp) exceeds 50% of both the query and truth variant lengths, meeting the minimum overlap threshold (--*min_ovl* = 0.5) (Equation (15)(16)).

overlap ≥ $L_{query}$× min_ovl	(15)

631 ≥ 631 × 0.5 $✓$

AND

overlap ≥ $L_{truth}$× min_ovl	(16)

631 ≥ 631 × 0.5 $✓$

As the truth and query deletion variants satisfy all evaluation criteria defined in EvalSVcallers—including chromosome and SVTYPE concordance, breakpoint distance tolerance, reciprocal overlap threshold—the deletion call in the query callset is classified as a true positive (TP). In contrast, because the truth set contains no insertion (INS) events, all insertion calls in the query callset are classified as false positives (FPs), while truth variants of the evaluated structural variant types that do not have a corresponding match in the query callset are classified as false negatives (FNs).

**SVbenchmark**

SVbenchmark is a comparison module within the SVanalyzer framework, designed to evaluate Structural Variant callsets (SVanalyzer Developers; https://svanalyzer.readthedocs.io/en/latest/svbenchmark.html). Evaluation begins with candidate-pair gating based on positional proximity using the --maxdist parameter (default: 100000 bp). A truth variant and a query variant are only considered comparable if the absolute difference between their start positions does not exceed this threshold as defined in Equation (17):

$\mid POS_{truth}-POS_{query}\mid\leq maxdist$	(17)

$$\mid130061662-130061662\mid\leq100000 ✓$$

Since this condition is satisfied, the deletion (Truth-DEL and Query-DEL) variants are accepted as a candidate pair.

For each candidate pair, SVbenchmark constructs an alternate haplotype implied by the truth variant and a separate alternate haplotype implied by the query variant by applying the corresponding alleles to the reference sequence. Importantly, each variant produces a single alternate haplotype regardless of genotype or haplotype configuration.

start = min(${truth}_{POS}$, ${query}_{POS}$) = $min\left( 130061662, 130061662 \right)=130061662$

end = max(${truth}_{END}$, ${query}_{END}$) =$max\left( 130062293, 130062293 \right)=130062293$

Since both variants have identical breakpoints and the same ALT (“C”), so the reconstructed alternate haplotypes are identical that implies:

$H_{1}$= alternate haplotype constructed from the truth variant

$H_{2}$= alternate haplotype constructed from the query variant

H_1_ = ref[start : ${truth}_{POS}$] + A_1_ + ref[${truth}_{END}$ : end]

= ref[$130061662:130061662]$ + “C” + ref[$130062293: 130062293]$ = C

H_2_ = ref[start : ${query}_{POS}$] + A_2_ + ref[${query}_{END}$ : end]

= ref[$130061662:130061662]$ + “C” + ref[$130062293: 130062293]$ = C

edit distance($H_{1}$, $H_{2}$) = 0

Once the alternate haplotypes are constructed, SVbenchmark defines the maximum affected region, which serves as the shared normalization denominator for all distance-based metrics. Maximum affected region is defined as the maximum of the lengths of the two constructed alternate haplotypes and represents the largest sequence region altered by either variant. All subsequent distance measures are normalized by this value to ensure scale-independent comparison across variants of different sizes.

The first metric evaluated is the maximum shift, which quantifies the absolute positional offset between the start coordinates of the truth and test variant effects (Equation (18)). This shift is normalized by the maximum affected region and must not exceed the *–normshift* threshold (default: 1.0) for the variants to remain compatible (Equation (19)(20)).

For each aligned base pair $(i_{k},j_{k})$ produced by the sequence alignment of $H_{1}$and $H_{2}$:

$i_{k}$= position of the base in $H_{1}$ = *C*

$j_{k}$= position of the corresponding base in $H_{2}$ = *C*

$maximum shift=\mid i_{k}-j_{k}\mid$	(18)

= ∣$130061662$−$130061662$∣ = 0

maximum affected region =$max(\mid H_{1}\mid, {\mid H}_{2}\mid)$	(19)

= max (1, 1) = 1

$$normalized shift distance=\frac{maximum shift}{maximum affected region} (20)$$

$$=\frac{0}{1}\leq1.0 ✓$$

The second metric is the size difference *–normsizediff* (default: 1.0), calculated as the absolute difference between the lengths of truth and query variants as defined in Equation (21). This value is likewise normalized by the maximum affected region.

$L_{truth}$= length of truth variant

$L_{query}$= length of query variant

$$normalized size difference=\frac{{|L}_{truth}-L_{query}|}{maximum affected region} (21)$$

$$=\frac{|631-631|}{1}\leq1.0 ✓$$

Finally, SVbenchmark computes the edit distance between the two alternate haplotypes. The edit distance is normalized by the maximum affected region and evaluated against the *--normdist* threshold (default: 1.0) as shown in Equation (22). Since H_1_ and H_2_ are identical, no edits are required.

$$normalized edit distance=\frac{edit distance}{maximum affected region} (22)$$

$$=\frac{0}{1}\leq1.0 ✓$$

A query–truth variant pair is classified as equivalent only if all three normalized metrics —maximum shift, size difference, and edit distance— simultaneously satisfy their respective thresholds, reflecting a strict logical AND decision rule. As in the preceding two frameworks, the truth set contains no insertion (INS) events resulting in all insertion variants in the query callset to be classified as false positives (FPs) and SV types in truth variants that do not have a corresponding match in the query callset are classified as false negatives (FNs).

**Command-Level Reproducibility Workflow**

To support command-level reproducibility, the complete computational workflow used in the revised study was deposited in the public GitHub repository and archived as a fixed release. The repository includes centralized configuration files, truth set and caller preprocessing scripts, framework specific baseline evaluation scripts, systematic parameter sensitivity configurations, genomic block resampling and paired variant level bootstrap workflows, baseline FP/FN characterization, cross-framework variant level analyses, figure and supplementary table generation scripts, software environment files, and repository level validation utilities. The principal repository components are summarized in Table S3. The revised workflow is available as GitHub release v1.1.0 and is permanently archived on Zenodo (DOI: 10.5281/zenodo.21903604).

**Table C.** Command-level reproducibility workflow and repository components.

| **Repository component** | **Purpose** | **Key content** |
| --- | --- | --- |
| scripts/00_config.sh | Centralized workflow configuration | Sample, reference, truth set, BAM/VCF, BED and output paths |
| scripts/run_callers.sh | SV calling workflow . | Manta, Delly, Lumpy/SVTyper, GRIDSS and Wham commands |
| scripts/01_preprocess_truth.sh | Truth set preprocessing | chr1–chr22, DEL/INS, \|SVLEN\| ≥50 bp; HG002 BED restriction |
| scripts/02_preprocess_callers.sh | Caller preprocessing | OctopuSV standardization, PASS, chr1–chr22, DEL/INS, \|SVLEN\| ≥50 bp |
| scripts/03–05_evaluate_*.sh | Baseline benchmarking | Truvari, EvalSVcallers and SVbenchmark default evaluations |
| configs/ | Sensitivity configurations | Truvari 17, SVbenchmark 17, EvalSVcallers 9 configurations |
| scripts/06_sensitivity.sh | OFAT sensitivity analysis | 430 sample–caller–framework–configuration evaluations |
| scripts/sensitivity_metrics/ | Sensitivity post-processing | Precision, Recall, F1 and baseline-relative ΔF1 |
| scripts/block_resampling/ | Genomic robustness analysis | 5-Mb blocks, 80% sampling without replacement, 200 iterations |
| scripts/variant_bootstrap/ | Parameter-change uncertainty | 5000 paired variant-level bootstrap iterations |
| scripts/error_characterization/ | Baseline FP/FN characterization | SV type- and size-stratified matched/unmatched analysis |
| scripts/cross_framework/ | Cross-framework variant analysis | TP agreement groups, breakpoint distance, size agreement and DEL overlap |
| scripts/analysis/ | Supplementary analyses | Preprocessing counts and supplementary-table generation |
| scripts/figures/ | Figure generation | Scripts used for revised analysis figures |
| envs/ | Software environments | Truvari, SVbenchmark, EvalSVcallers and analysis Conda environments |
| scripts/preflight_repository.sh | Repository consistency checks | Configuration, path and parameter checks |
| scripts/check_syntax_and_dependencies.sh | Code validation | Python/Bash syntax and dependency checks |
| archive_reference/ | Final reference tables | Archived manuscript-supporting CSV tables |
| GitHub/Zenodo release | Fixed reproducibility snapsho | v1.1.0; commit 889fda3; DOI 10.5281/zenodo.21903604 |

**Table D.** Variant counts across preprocessing steps.

| **Sample** | **Caller** | **Raw input*** | **Raw BND*** | **BND resolved*** | **After OctopuSV*** | **DEL/INS*** | **chr1–22*** | **≥50 bp*** | **Final*** |
| --- | --- | --- | --- | --- | --- | --- | --- | --- | --- |
| HG002 | Manta | 10464 | 1118 | 950 | 10171 | 8699 | 8433 | 7737 | 7238 |
| HG002 | Delly | 20823 | 8480 | 8480 | 20823 | 9373 | 9007 | 2495 | 1751 |
| HG002 | Lumpy | 5449 | 3312 | 3312 | 4466 | 2033 | 1902 | 1902 | 1081 |
| HG002 | GRIDSS | 825046 | 825046 | 813614 | 272472 | 24626 | 24078 | 3337 | 3147 |
| HG002 | Wham | 4913 | 0 | 0 | 4913 | 4905 | 4652 | 2559 | 2074 |
| NA12878 | Manta | 12214 | 3490 | 3264 | 11306 | 7788 | 7509 | 7080 | 7080 |
| NA12878 | Delly | 147447 | 100631 | 100631 | 147445 | 14880 | 13857 | 7426 | 7426 |
| NA12878 | Lumpy | 27264 | 17940 | 17940 | 22066 | 9153 | 8253 | 5963 | 5963 |
| NA12878 | GRIDSS | 1036324 | 1036324 | 1025278 | 333629 | 28405 | 27535 | 3405 | 3405 |
| NA12878 | Wham | 9203 | 0 | 0 | 9203 | 6988 | 6183 | 3213 | 3213 |

*Raw caller VCF record counts, BND records before representation standardization, BND records no longer represented as BND following OctopuSV v0.3.1, total records after OctopuSV, and the numbers retained after subsequent DEL/INS extraction, autosomal restriction, and minimum-size filtering are shown for each sample–caller combination. For HG002, the final benchmarking set additionally reflects restriction to the corresponding high-confidence BED regions. No equivalent BED restriction was applied to NA12878.

**Table E.** SV-size distribution according to cross-framework TP agreement.

| **Sample** | **Caller** | **SVTYPE** | **TP agreement** | **Total** | **50–99 bp** | **100–499 bp** | **500–999 bp** | **1–9.9 kb** | **≥10 kb** |
| --- | --- | --- | --- | --- | --- | --- | --- | --- | --- |
| HG002 | Manta | DEL | All three | 3985 | 1378 (34.6%) | 1850 (46.4%) | 170 (4.3%) | 553 (13.9%) | 34 (0.9%) |
| HG002 | Manta | DEL | Two frameworks | 102 | 26 (25.5%) | 62 (60.8%) | 4 (3.9%) | 9 (8.8%) | 1 (1.0%) |
| HG002 | Manta | DEL | One framework | 33 | 10 (30.3%) | 15 (45.5%) | 6 (18.2%) | 2 (6.1%) | 0 (0.0%) |
| HG002 | Manta | INS | All three | 2468 | 1261 (51.1%) | 1129 (45.7%) | 78 (3.2%) | 0 (0.0%) | 0 (0.0%) |
| HG002 | Manta | INS | Two frameworks | 146 | 69 (47.3%) | 73 (50.0%) | 4 (2.7%) | 0 (0.0%) | 0 (0.0%) |
| HG002 | Manta | INS | One framework | 60 | 15 (25.0%) | 25 (41.7%) | 10 (16.7%) | 10 (16.7%) | 0 (0.0%) |
| HG002 | Delly | DEL | Two frameworks | 1080 | 323 (29.9%) | 1 (0.1%) | 139 (12.9%) | 580 (53.7%) | 37 (3.4%) |
| HG002 | Delly | DEL | One framework | 1325 | 330 (24.9%) | 2 (0.2%) | 236 (17.8%) | 713 (53.8%) | 44 (3.3%) |
| HG002 | Delly | INS | Two frameworks | 2 | 2 (100.0%) | 0 (0.0%) | 0 (0.0%) | 0 (0.0%) | 0 (0.0%) |
| HG002 | Delly | INS | One framework | 69 | 0 (0.0%) | 16 (23.2%) | 34 (49.3%) | 17 (24.6%) | 2 (2.9%) |
| HG002 | Lumpy | DEL | All three | 685 | 0 (0.0%) | 0 (0.0%) | 56 (8.2%) | 591 (86.3%) | 38 (5.5%) |
| HG002 | Lumpy | DEL | Two frameworks | 51 | 0 (0.0%) | 0 (0.0%) | 4 (7.8%) | 45 (88.2%) | 2 (3.9%) |
| HG002 | Lumpy | DEL | One framework | 12 | 0 (0.0%) | 0 (0.0%) | 0 (0.0%) | 11 (91.7%) | 1 (8.3%) |
| HG002 | Lumpy | INS | One framework | 51 | 0 (0.0%) | 8 (15.7%) | 23 (45.1%) | 18 (35.3%) | 2 (3.9%) |
| HG002 | GRIDSS | DEL | All three | 2467 | 481 (19.5%) | 1358 (55.0%) | 149 (6.0%) | 451 (18.3%) | 28 (1.1%) |
| HG002 | GRIDSS | DEL | Two frameworks | 18 | 7 (38.9%) | 4 (22.2%) | 3 (16.7%) | 3 (16.7%) | 1 (5.6%) |
| HG002 | GRIDSS | DEL | One framework | 4 | 1 (25.0%) | 0 (0.0%) | 2 (50.0%) | 1 (25.0%) | 0 (0.0%) |
| HG002 | GRIDSS | INS | Two frameworks | 2 | 1 (50.0%) | 1 (50.0%) | 0 (0.0%) | 0 (0.0%) | 0 (0.0%) |
| HG002 | GRIDSS | INS | One framework | 426 | 307 (72.1%) | 100 (23.5%) | 3 (0.7%) | 14 (3.3%) | 2 (0.5%) |
| HG002 | Wham | DEL | All three | 8 | 0 (0.0%) | 1 (12.5%) | 5 (62.5%) | 2 (25.0%) | 0 (0.0%) |
| HG002 | Wham | DEL | Two frameworks | 43 | 3 (7.0%) | 5 (11.6%) | 30 (69.8%) | 5 (11.6%) | 0 (0.0%) |
| HG002 | Wham | DEL | One framework | 260 | 1 (0.4%) | 20 (7.7%) | 237 (91.2%) | 2 (0.8%) | 0 (0.0%) |
| HG002 | Wham | INS | Two frameworks | 3 | 1 (33.3%) | 2 (66.7%) | 0 (0.0%) | 0 (0.0%) | 0 (0.0%) |
| HG002 | Wham | INS | One framework | 1015 | 343 (33.8%) | 669 (65.9%) | 2 (0.2%) | 1 (0.1%) | 0 (0.0%) |
| NA12878 | Manta | DEL | All three | 1750 | 529 (30.2%) | 938 (53.6%) | 58 (3.3%) | 215 (12.3%) | 10 (0.6%) |
| NA12878 | Manta | DEL | Two frameworks | 103 | 40 (38.8%) | 53 (51.5%) | 1 (1.0%) | 9 (8.7%) | 0 (0.0%) |
| NA12878 | Manta | DEL | One framework | 23 | 6 (26.1%) | 10 (43.5%) | 3 (13.0%) | 3 (13.0%) | 1 (4.3%) |
| NA12878 | Manta | INS | All three | 1192 | 545 (45.7%) | 622 (52.2%) | 25 (2.1%) | 0 (0.0%) | 0 (0.0%) |
| NA12878 | Manta | INS | Two frameworks | 173 | 96 (55.5%) | 72 (41.6%) | 5 (2.9%) | 0 (0.0%) | 0 (0.0%) |
| NA12878 | Manta | INS | One framework | 187 | 97 (51.9%) | 79 (42.2%) | 4 (2.1%) | 6 (3.2%) | 1 (0.5%) |
| NA12878 | Delly | DEL | Two frameworks | 1288 | 198 (15.4%) | 775 (60.2%) | 71 (5.5%) | 233 (18.1%) | 11 (0.9%) |
| NA12878 | Delly | DEL | One framework | 1446 | 219 (15.1%) | 793 (54.8%) | 116 (8.0%) | 300 (20.7%) | 18 (1.2%) |
| NA12878 | Delly | INS | Two frameworks | 78 | 78 (100.0%) | 0 (0.0%) | 0 (0.0%) | 0 (0.0%) | 0 (0.0%) |
| NA12878 | Delly | INS | One framework | 95 | 0 (0.0%) | 59 (62.1%) | 20 (21.1%) | 12 (12.6%) | 4 (4.2%) |
| NA12878 | Lumpy | DEL | All three | 1162 | 98 (8.4%) | 750 (64.5%) | 74 (6.4%) | 228 (19.6%) | 12 (1.0%) |
| NA12878 | Lumpy | DEL | Two frameworks | 107 | 45 (42.1%) | 26 (24.3%) | 14 (13.1%) | 22 (20.6%) | 0 (0.0%) |
| NA12878 | Lumpy | DEL | One framework | 39 | 1 (2.6%) | 3 (7.7%) | 12 (30.8%) | 19 (48.7%) | 4 (10.3%) |
| NA12878 | Lumpy | INS | One framework | 113 | 0 (0.0%) | 76 (67.3%) | 19 (16.8%) | 15 (13.3%) | 3 (2.7%) |
| NA12878 | GRIDSS | DEL | All three | 1220 | 215 (17.6%) | 764 (62.6%) | 58 (4.8%) | 173 (14.2%) | 10 (0.8%) |
| NA12878 | GRIDSS | DEL | Two frameworks | 36 | 12 (33.3%) | 17 (47.2%) | 2 (5.6%) | 5 (13.9%) | 0 (0.0%) |
| NA12878 | GRIDSS | DEL | One framework | 3 | 1 (33.3%) | 0 (0.0%) | 0 (0.0%) | 0 (0.0%) | 2 (66.7%) |
| NA12878 | GRIDSS | INS | Two frameworks | 3 | 3 (100.0%) | 0 (0.0%) | 0 (0.0%) | 0 (0.0%) | 0 (0.0%) |
| NA12878 | GRIDSS | INS | One framework | 293 | 209 (71.3%) | 74 (25.3%) | 1 (0.3%) | 7 (2.4%) | 2 (0.7%) |
| NA12878 | Wham | DEL | All three | 533 | 39 (7.3%) | 281 (52.7%) | 48 (9.0%) | 157 (29.5%) | 8 (1.5%) |
| NA12878 | Wham | DEL | Two frameworks | 34 | 0 (0.0%) | 3 (8.8%) | 1 (2.9%) | 30 (88.2%) | 0 (0.0%) |
| NA12878 | Wham | DEL | One framework | 12 | 1 (8.3%) | 2 (16.7%) | 3 (25.0%) | 5 (41.7%) | 1 (8.3%) |
| NA12878 | Wham | INS | Two frameworks | 3 | 3 (100.0%) | 0 (0.0%) | 0 (0.0%) | 0 (0.0%) | 0 (0.0%) |
| NA12878 | Wham | INS | One framework | 370 | 229 (61.9%) | 134 (36.2%) | 2 (0.5%) | 5 (1.4%) | 0 (0.0%) |

**Fig A.** Caller-side baseline FP burden by SV type and size.

**Fig B.** Truth-side baseline FN burden by SV type and size.

**Fig C.** Overlap of TP variants across frameworks for HG002.

**Fig D.** Overlap of TP variants across frameworks for NA12878.

**Fig E.** Pairwise Jaccard index and overlap coefficient of TP variants across frameworks for HG002.

**Fig F.** Pairwise Jaccard index and overlap coefficient of TP variants across frameworks for NA12878.

**Fig G.** Sankey diagram showing the flow of labels (TP, FP, NONE) across frameworks for HG002.

**Fig H.** Sankey diagram showing the flow of labels (TP, FP, NONE) across frameworks for NA12878.

**Fig I.** DEL characteristics by cross-framework TP agreement.

**Fig J.** INS characteristics by cross-framework TP agreement.

**References**

English AC, Menon VK, Gibbs RA, Metcalf GA, Sedlazeck FJ. Truvari: refined structural variant comparison preserves allelic diversity. Genome Biol. 2022 Dec 27;23(1):271. doi: 10.1186/s13059-022-02840-6. PMID: 36575487; PMCID: PMC9793516.

ACEnglish. Truvari: Toolkit for benchmarking, merging, and annotating Structural Variants. Available at: https://github.com/acenglish/truvari (Accessed: 05 January 2026).

Kosugi S, Momozawa Y, Liu X, Terao C, Kubo M, Kamatani Y. Comprehensive evaluation of structural variation detection algorithms for whole genome sequencing. Genome Biology 20(1):117 (2019). doi: 10.1186/s13059-019-1720-5.

stat-lab. EvalSVcallers. Available at: https://github.com/stat-lab/EvalSVcallers (Accessed: 05 January 2026).

SVanalyzer Developers. SVbenchmark: Structural variant benchmarking module. Available at: https://svanalyzer.readthedocs.io/en/latest/svbenchmark.html#svbenchmark (Accessed: 05 January 2026).
